# A Module of Human Peripheral Blood Mononuclear Cell Transcriptional Network Containing Primitive and Differentiation Markers Is Related to Specific Cardiovascular Health Variables

**DOI:** 10.1371/journal.pone.0095124

**Published:** 2014-04-23

**Authors:** Leni Moldovan, Mirela Anghelina, Taylor Kantor, Desiree Jones, Enass Ramadan, Yang Xiang, Kun Huang, Arunark Kolipaka, William Malarkey, Nima Ghasemzadeh, Peter J. Mohler, Arshed Quyyumi, Nicanor I. Moldovan

**Affiliations:** 1 Davis Heart and Lung Research Institute, Wexner Medical Center, The Ohio State University, Columbus, Ohio, United States of America; 2 Department of Internal Medicine, Wexner Medical Center, The Ohio State University, Columbus, Ohio, United States of America; 3 Department of Biomedical Informatics, Wexner Medical Center, The Ohio State University, Columbus, Ohio, United States of America; 4 Cardiovascular Clinical Research Institute, Emory University, Atlanta, Georgia, United States of America; Emory University School of Medicine, United States of America

## Abstract

Peripheral blood mononuclear cells (PBMCs), including rare circulating stem and progenitor cells (CSPCs), have important yet poorly understood roles in the maintenance and repair of blood vessels and perfused organs. Our hypothesis was that the identities and functions of CSPCs in cardiovascular health could be ascertained by analyzing the patterns of their co-expressed markers in unselected PBMC samples. Because gene microarrays had failed to detect many stem cell-associated genes, we performed quantitative real-time PCR to measure the expression of 45 primitive and tissue differentiation markers in PBMCs from healthy and hypertensive human subjects. We compared these expression levels to the subjects' demographic and cardiovascular risk factors, including vascular stiffness. The tested marker genes were expressed in all of samples and organized in hierarchical transcriptional network modules, constructed by a bottom-up approach. An index of gene expression in one of these modules (metagene), defined as the average standardized relative copy numbers of 15 pluripotency and cardiovascular differentiation markers, was negatively correlated (all p<0.03) with age (R^2^ = −0.23), vascular stiffness (R^2^ = −0.24), and central aortic pressure (R^2^ = −0.19) and positively correlated with body mass index (R^2^ = 0.72, in women). The co-expression of three neovascular markers was validated at the single-cell level using mRNA *in situ* hybridization and immunocytochemistry. The overall gene expression in this cardiovascular module was reduced by 72±22% in the patients compared with controls. However, the compactness of both modules was increased in the patients' samples, which was reflected in reduced dispersion of their nodes' degrees of connectivity, suggesting a more primitive character of the patients' CSPCs. In conclusion, our results show that the relationship between CSPCs and vascular function is encoded in modules of the PBMCs transcriptional network. Furthermore, the coordinated gene expression in these modules can be linked to cardiovascular risk factors and subclinical cardiovascular disease; thus, this measure may be useful for their diagnosis and prognosis.

## Introduction

Circulating stem/progenitor cells (CSPCs) contribute to the maintenance of the normal functions of blood vessels and tissues and their repair and regeneration [1]. These cells may also promote tumor growth by facilitating neovascularization or the development of tumor stroma [2]. CSPCs and other leukocytes mediate these actions through the release of paracrine factors [3] and occasionally by transdifferentiation [4]. The numbers and functions of CSPCs are impaired by exposure to cardiovascular risk factors, such as aging, diabetes, hyperlipidemia, or hypertension (for a review, see [5]). Moreover, the frequency of CSPCs was inversely related to subclinical vascular diseases, including endothelial dysfunction and arterial stiffness [6].

A major obstacle to progress in this field has been a lack of consensus regarding the precise molecular markers that define these regenerative pathways [7]. This problem is compounded by the limited accuracy and reproducibility of the current methods used to quantitate CSPCs, such as flow cytometry [8] and *in vitro* colony formation assays for ‘early’ [9] or ‘late’ [10] progenitor cells. Potential novel tools that may be used to address these issues include the emerging network sciences as applied to biology and medicine [11].

Transcription progressively enables primitive cells to acquire a differentiated phenotype [12], whereas the expression of primitive genes is indicative of cell stemness in both bone marrow and blood [13]. However, the reason for the presence of mRNA for tissue-specific differentiation genes in circulating cells is less clear. Because more than 80% of the genes expressed in human peripheral blood are also expressed in other body tissues [14], mRNA profiling of leukocytes has been proposed as an accessible window to the multi-organ transcriptome [15]. Additionally, the transcriptional landscape, including those of adult hematopoietic stem cells and adult leukocytes, is organized as a modular network of co-expressed genes [16]. Cardiovascular disease-associated transcriptomic signatures are known to exist in peripheral blood [17]; however, none has yet been found to specifically contain CSPC markers or be directly relevant to vascular function in healthy subjects.

Our hypothesis was that the origins of primitive and tissue-specific mRNAs in peripheral blood mononuclear cell (PBMC) samples would be primarily, although not exclusively, in CSPCs. If supported by data, then the coordinated expression of CSPC-derived mRNAs should be detectable in peripheral blood transcriptional profiles and reflect the function of the corresponding tissues, similar to the actual tissue-specific CSPCs. Here, we developed and functionally validated such a method, which applies network science to transcriptomic analyses.

Because the high-throughput charting of a transcriptome either produces many irrelevant hits or is often too insensitive for specific targets [18], predesigned gene panels are increasingly used for the detection of gene expression signatures in tissues [19] and the assessment of pluripotency [20] or differentiation hierarchy in stem cells [21]. To detect rare transcripts, the most reliable technique to date remains quantitative real-time PCR (qRT-PCR), which is accurate, precise, more sensitive than microarrays, and more specific for mature transcripts than RNA sequencing [18]. qRT-PCR has been used to generate transcriptional networks from as few as 18 transcription factors [22] to as many as 280 of the ‘most-used’ hand-picked stem cell markers [21]. The transcriptional signatures of individual CSPC-associated markers have been previously detected using qRT-PCR in human and animal peripheral blood at baseline and after major cardiovascular and neurovascular events [23].

As reported here, microarrays (specifically, Affymetrix GeneChips) were unable to detect many of the stem cell-related genes in PBMCs samples, a finding that confirmed previous studies [21]. Thus, to create a sensitive and efficient assay, we designed a gene panel that included the most-recognized stem/progenitor cell and tissue differentiation markers (Table S1). These markers were simultaneously measured in PBMCs using qRT-PCR and then subjected to a network analysis and validated for vascular function.

## Methods

### Subjects

Institutional Review Board (IRB) approvals were obtained from The Ohio State University and Emory University, and all subjects signed written informed consent forms. We recruited 26 healthy volunteers at Ohio State University and 20 hypertensive subjects at Emory University (Table 1 and Table 2, respectively).

**Table 1:** Characteristics of the control subjects.

Parameter	Value	Percent/range
Gender		
Males	12	46.2%
Females	14	53.8%
Race		
White	16	61.5%
African American	7	27.0%
Asian	3	11.5%
Age, years	40.6±11.6	19–58
Height (cm)	171.2±11.4	152–193
Weight (kg)	83.2±21.2	60–136
BMI	28.7±8.1	19.2–53.13
Seated SBP (mm Hg)	113.7±11.6	95–140
Seated DBP (mm Hg)	74.5±7.9	58–87
Heart rate (bmp)	68.7±10.7	52–100
AIx	17.4±12.4	(−8)–43
AoPP	29.8±8.2	19–50
AoSP	104.8±10.5	87–124

**Table 2:** Characteristics of the hypertensive subjects.

Parameter	Value	Percent/range
Gender		
Males	14	70%
Females	6	30%
Race		
White	8	40%
African American	11	55%
Hispanic	1	5%
Age, years	65.7±11.3	41–85
Height (cm)	171.4±8.4	155–188
Weight (kg)	84.7±20.7	51–118
BMI	28.8±6.8	20–39
Seated SBP (mm Hg)	149.0±21.9	116–194
Seated DBP (mm Hg)	80.0±10.5	63–99
Heart rate (bpm)	73.3±12.8	56–102

### Blood pressure and pulse wave measurements

Brachial blood pressure (BP) and radial pulse wave values were measured in the healthy subjects using a manual oscillometric monitor with a standard adult cuff and the SphygmoCor device (AtCor Medical, Sydney, Australia), respectively. The latter provides a validated generalized transfer function to convert the peripheral radial arterial pulse wave into the equivalent central aortic arterial pulse wave, which was used to analyze the derived aortic pressure waveform and extract the aortic pulse pressure (AoPP) and the augmentation index (AIx) of the pulse, a surrogate measure of vascular stiffness [24].

### Magnetic resonance elastography (MRE)

MRE is a magnetic resonance imaging (MRI)-based method that directly determines organ stiffness and has been validated in a study of hypertensive patients [25]. In aortic MRE, vibrations are applied to the abdomen by an electro-mechanical driver, which transmits mechanical waves to the aorta. A phase-contrast MRI sequence synchronized to externally applied vibrations measures the wave displacement field, which is mathematically converted to stiffness [26].

### Isolation of PBMCs

Blood was collected into a BD Vacutainer K_2_ EDTA (BD Bioscience, Franklin Lakes, NJ), diluted 1∶1 with Hanks balanced salt solution (HBSS) (Invitrogen Life Technologies, Grand Island, NY), layered onto one volume of Lymphocyte Separation Medium (Cellgro Mediatech Inc., Manassas, VA), and centrifuged at 700×g for 30 min at room temperature. The mononuclear cells were collected, diluted 1∶1 with washing buffer (PBS supplemented with 2 mM EDTA and 2% FBS) (Gemini Bio-Products, West Sacramento, CA), and centrifuged at 300×g for 7 min. The pellet was resuspended in 5 mL of 0.8% ammonium chloride solution (STEMCELL Technologies Inc., Vancouver, Canada) for 5 min to lyse any remaining erythrocytes. To remove as many platelets as possible, the cells were washed two more times as described above with two volumes of washing buffer.

### RNA extraction and qRT-PCR

Total RNA was isolated using the RNeasy Mini Kit (Qiagen, Valencia, CA) according to the manufacturer's protocol, tested for quality, and stored at −80°C until use. The VILO kit (Life Technologies/Invitrogen, Grand Island, NY) was used to reverse transcribe 150 ng of total RNA. Primers (Qiagen) were diluted 1∶20 with molecular-grade water, and 5 µL/well were added to 384-well plates using a Biomek FX Laboratory Automation Workstation (Beckman Coulter, Inc., Brea, CA). The plates were left to dry overnight in a sterile hood and stored covered at −20°C until use. qRT-PCR was performed using SYBR Green (Qiagen) and a 7900HT Real-Time PCR System (Life Technologies/Applied Biosystems, Foster City, CA) operated in standard mode. All of the runs contained a dissociation step. The samples were amplified in duplicate in a total volume of 5 µL. The results are expressed as the relative copy number (RCN), defined as RCN = 2^−ΔCq^×100, where ΔCq is the difference Cq(target) – Cq(reference) [27]. As a reference for normalization, we used the median Cq values of three endogenous controls (beta-2 microglobulin, GAPDH and RPL13). Data were were uploaded to Gene Expression Omnibus (GSE56327).

### Fluorescence RNA *in situ* hybridization (ISH)

Cells (up to 1×10^6^) from eight volunteers were fixed in suspension (4% formaldehyde in PBS for 1 h) and deposited onto Superfrost Plus microscope slides (Fisher Scientific, Pittsburg, PA) by cytospin centrifugation (180×g, 5 min) in a Cytospin 2 Shandon Centrifuge (Block Scientific, Inc., Bohemia, NY). The slides were dried at 37°C for 1 h and stored in 100% ethanol at −80°C until use. ISH was performed using the QuantiGene ViewRNA kit (Affymetrix/Panomics Solutions, Santa Clara, CA) according to the manufacturer's protocol with the following probe sets: FSHR-FITC, NES-Cy3, and KDR-Cy5, and the nuclei were counterstained with DAPI (Sigma-Aldrich, St. Louis, MO). In preliminary tests, we found that a 1∶1000 dilution of proteinase K was optimal. The samples were analyzed with an Olympus FB1000 confocal microscope (Olympus America Inc., Melville, NY). The 40x objective and 3x zoom were used to acquire Z-stacks from 5-8 fields per sample, for a total of 100–600 cells per subject. The acquisition settings were adjusted based on the negative control slides (no hybridization probe). The images were analyzed with CellProfiler 2.0 software [28] (http://www.cellprofiler.org/), as follows. (i) Using FluoView software (Olympus), all of the images for each Z-stack per each channel were exported as .tiff files. (ii) Using MetaMorph software (Molecular Devices, Sunnyvale, CA), all of the images from the Z-stacks were assembled into a separate .stk file for each channel. (iii) Using CellProfiler, we built four pipelines, one for each channel (blue: DAPI/nuclei, green: FSHR-FITC, red: NES-Cy3, and cyan: KDR-Cy5), which uploaded the respective stacks and generated a projection. Steps 1–3 were necessary because the CellProfiler software could only recognize MetaMorph stacks. (iv) Next, we built a pipeline for the analysis of these projections. An important step was the setting of thresholds. To detect nuclei (‘primary objects’), we entered a valid range of diameters (in pixels), and the threshold was set such that >95% of nuclei (including clumped nuclei) were detected accurately. Cell boundaries (‘secondary objects’) were estimated by moving 10 pixels outward from the cell nucleus (Distance-N method). For RNA quantification, the thresholds for each channel were set individually based on negative controls. We measured spot numbers, spot intensities, and a variety of nuclear parameters (such as intensity, area, and texture) offered by the software. (v) Finally, the data were exported into Excel and pooled from all of the images/subjects. At this point, we manually removed all nuclear objects that were inaccurately detected such as clumps that could not be segmented into individual nuclei and small fragments that clearly corresponded to cellular debris based on size (the ‘nuclear area’ parameter). Finally, each cell (and its associated measurements) was given a unique ID. All of the subsequent statistical analyses and visualizations were performed using various statistical packages (*vide infra*).

### Immunocytochemistry (ICC)

For ICC, PBMCs from four subjects were fixed in 1% formaldehyde in PBS for 1 h, cytospun as described, and used immediately. The cells were permeabilized with Triton X-100 (Fisher Biotech, Pittsburgh, PA) for 10 min at room temperature and blocked with 5% BSA + FcR Binding Inhibitor Purified (eBioscience, Inc., San Diego, CA) for 30 min at room temperature. The cells were incubated sequentially with the following antibodies: (i) rabbit polyclonal anti-FSHR antibody; (ii) Alexa Fluor-594 goat anti-rabbit antibody; and (iii) Alexa Fluor-488 monoclonal anti-human nestin antibody (Abcam, Cambridge, MA), along with Alexa Fluor-647 mouse anti-human CD309 (BD Pharmingen, San Jose, CA). DAPI was used as a nuclear stain, and the cells were mounted in Fluoromount-G (SouthernBiotech, Birmingham, Al). Alexa Fluor-488, Alexa Fluor-647 mouse IgG 1 isotype, and Alexa Fluor-594 goat anti-rabbit IgG (BD) were used as controls. The preparations were imaged with an Olympus FV 1000 confocal microscope using the 40x objective and 2x zoom, and negative controls were used for setting the lasers as described for ISH. We acquired 5–6 random images per slide, for a total of 300–700 cells per subject. In each fraction of cells (including most of those that were single- and double-positive for KDR and/or FSHR), NES and FSHR were localized to a few, usually 1–4, compact circular structures that were intimately associated with the nucleus or nuclear grooves (possibly representing spurious cross-reactions of our antibodies with either storage or degradation compartments). Therefore, we excluded these features from quantification. The images were analyzed with the CellProfiler software using a different pipeline. To adjust the threshold correction factors for each channel, we used the negative controls. The data were exported into Excel and further processed as described for ViewRNA, step 5. Both pipelines were uploaded on the CellProfiler website as VR-3Plex-FNK.cp and ICC-NFK.cp, respectively.

### *In silico* gene expression analysis

Studies were selected from the National Center for Biotechnology Information's Gene Expression Omnibus (GEO) according to the following criteria: (i) they employed Affymetrix Human Genome U133 Plus 2.0 Arrays (platform GPL570); (ii) they used isolated PBMCs; and (iii) they included healthy subjects as controls. We chose eight studies (GSE: 8507, 10041, 11761, 14642, 19743, 21942, 27034, and 46480) and only included the GSM files that corresponded to controls in the analysis (a total of 274 GeneChips), and a study on isolated bone marrow-derived CD34^+^ cells (GSE 23025) [29]. All of the .cel files were imported into Expression Console (Affymetrix), and we used the MAS5 algorithm for normalization and signal and presence call detection. We then extracted the information pertaining to the genes of interest. For genes represented by several probe sets on the array, we calculated the median signal value for each probe set over all the arrays and retained the sets that had the highest value. Finally, the ‘Present’, ‘Marginal’, and ‘Absent’ calls were recorded for each gene, and the ‘Presence Score’ was calculated as a percentage of all 274 arrays. Because genes with ‘Marginal’ scores were found only on a small number of arrays, all of these were considered ‘Absent’.

### Pattern analysis and network visualization

To mine patterns from the gene co-expression matrix data, we followed the network mining and merging workflow described by Xiang *et al*. [30]. First, we converted a gene co-expression dataset into a unweighted graph by creating an edge between any two genes with an absolute correlation value greater than 0.7. After the graph was created, we applied the Bron-Kerbosch algorithm [31] to generate all of the maximal cliques. We then applied the network merge approach [30] to these cliques under a density threshold 0.8, which guaranteed that each resulting sub-network induced a sub-matrix with an average correlation value greater than 0.8 on the original gene co-expression matrix. Finally, we visualized the discovered sub-networks using Gephi [32] (www.gephi.org).

### Data analysis

ANOVA, t-test, Mann-Whitney test, and various correlation statistics (Pearson correlation, linear regression, principal component analysis, hierarchical clustering, Cronbach's alpha) were performed using JMP 10.0.2 (SAS Institute, Inc., Cary, NC), Partek Discovery Suite v. 6.4 (Partek Inc., St. Louis, MO), and Microsoft Excel 2010 programs. In all of the statistical analyses, p<0.05 was considered significant.

## Results

### Limitations of the microarrays in detecting mRNAs with low expression levels

We first attempted to determine the transcriptional signature of CSPCs in blood using the Affymetrix GeneChips, as expression levels of a panel of the most common stem/progenitor cell and differentiation marker genes (Table S1, with annotations). We analyzed a set of 274 microarrays compounded from public databases, representing 7 studies of PBMCs and one study of purified CD34^+^ stem cells, all from healthy human subjects. Gene detection, determined as a ‘Present’ call by the MAS5 algorithm, varied as follows. (i) Other than housekeeping genes (B2M, GAPDH, and RPL13), only leukocyte genes (CD14, CD79A, CD3E, ITGAM, PECAM1, NT5E/CD73, and PTPRC/CD45) and a few others (ACTA2, ALDH1A1, BGLAP/osteocalcin, CX3CR1, and CXCR4) had a ‘Present’ call on more than 90% of the arrays. (ii) Several primitive (GATA4, NANOG, NES, NKX2-5, POUF5F1/OCT4, and THY1) and differentiation (CAV3, CDH5, CNN1, FSHR, KRT14, NOS3, and TEK/TIE2) genes were completely undetected (i.e., 0% ‘Present’ call). (iii) The other tested genes had a variable representation on the arrays (Table S2). Surprisingly, even the mRNA for CD34, the marker for which the cell suspension had been enriched using magnetic immunoselection, was detected on only 80% of the arrays. These findings confirmed the low sensitivity and/or poor reliability of the GeneChip microarrays for the detection of rare transcripts, as previously noted [33].

### Quantification by qRT-PCR and organization of a PBMC transcriptional sub-system

**Figure 1:**
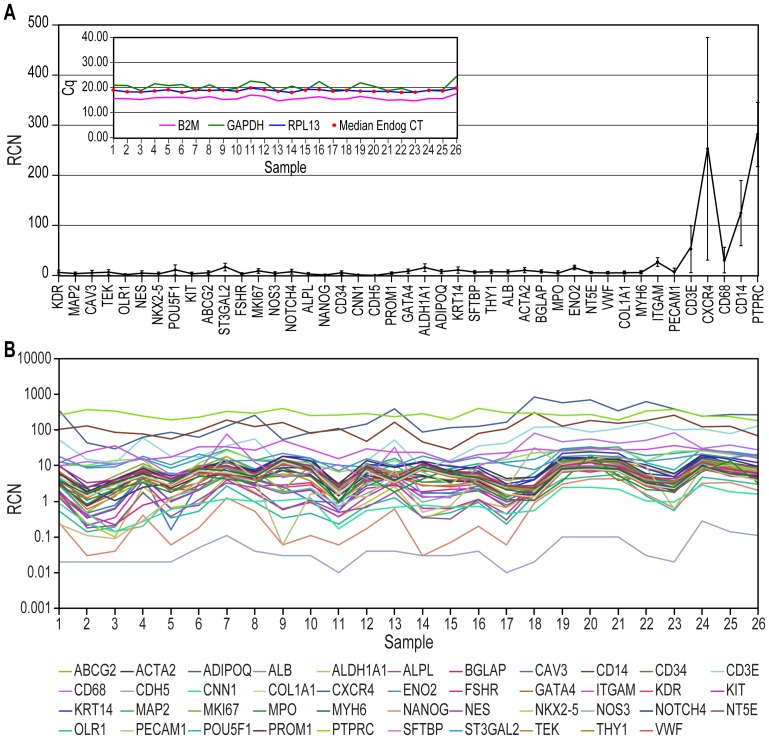
qRT-PCR quantification of gene expression in PBMCs from a sample of a healthy human population. **A**. The average expression levels (indicated as the relative copy number, RCN = 2^−ΔCq^×100) of the tested genes ordered based on the strength of their covariation (compare to Fig. 2A). The data are expressed as the means ± SD. *Inset*. Cq values for housekeeping genes used as endogenous controls. Of note, the large SD displayed by CXCR4 was due not to outliers but to the skewness of the data distribution. **B**. Actual RCN values of the 45 tested genes in 26 healthy subjects, indicating the coordinated expression of the majority of the genes (conventional color coding).

**Figure 2:**
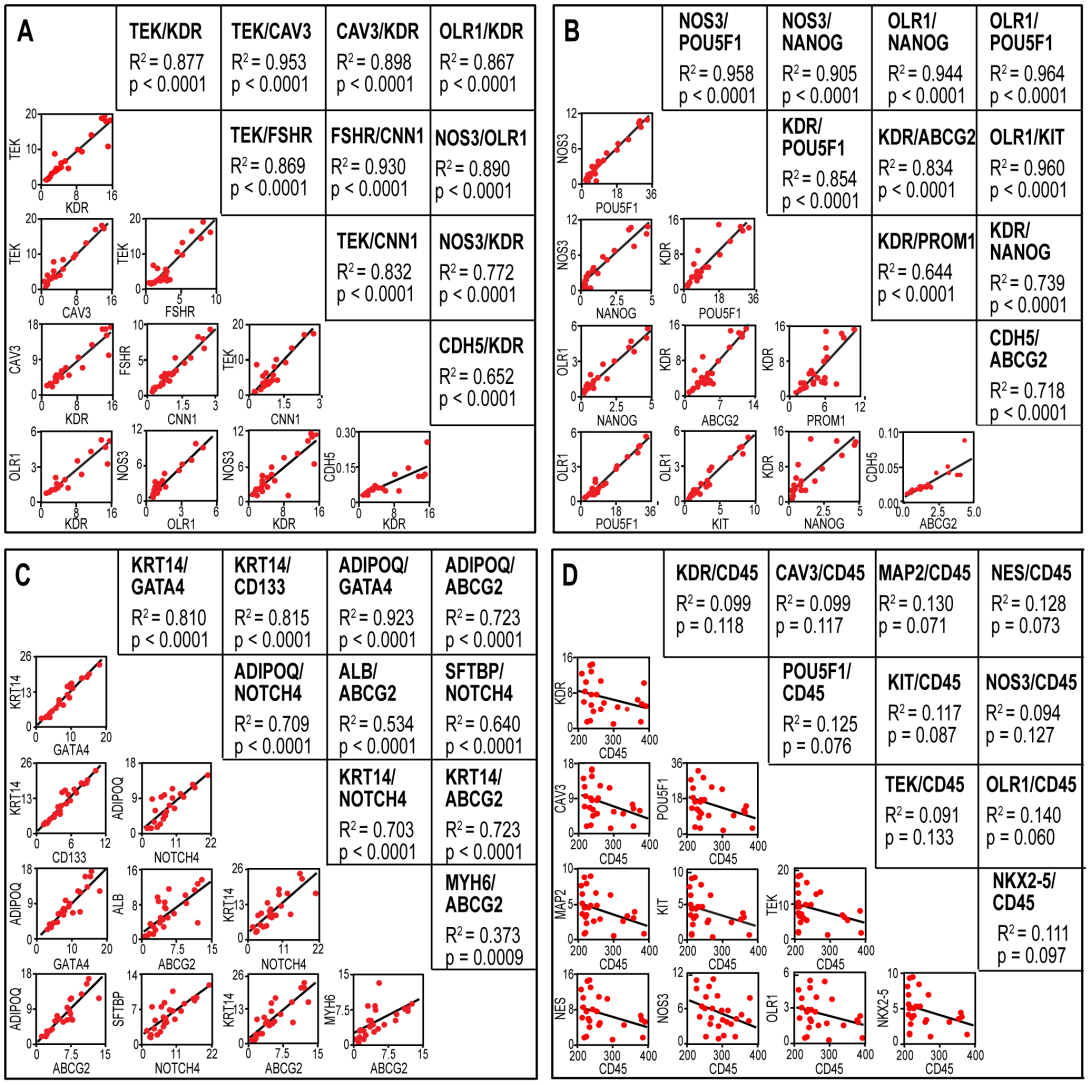
Regression analysis of genes co-expressed in PBMCs isolated from healthy human adults. **A**. Correlations between several cardiovascular-specific genes. **B**. Correlations between selected vascular and primitive genes in the same population. **C**. Correlations between other tissue-specific and primitive genes. **D**. Inverse correlation between the expression of vascular genes and a leukocyte gene (PTPRC/CD45). In all the graphs, the number of subjects is n = 26, R^2^ is the linear regression coefficient, and p indicates significance; these coefficients were placed in mirror positions across the diagonal with their corresponding graphs. The data represent RCNs.

In contrast, all of the tested mRNAs were detected in PBMCs from all of the healthy adults using qRT-PCR (Fig. 1 and Table S1). Genes with the smallest relative copy numbers (RCNs) represented the primitive and tissue-specific (including cardiovascular) genes, whereas the leukocyte-associated genes had much larger RCNs (Fig. 1A). We also observed a pattern suggestive of covariation between many of the mRNA markers (Fig. 1B). Regression analysis demonstrated that indeed the expression levels of cardiovascular genes were highly correlated with each other and with those of several primitive genes (Fig. 2A and 2B, respectively). A strong covariation was demonstrated as well between several tissue-associated genes and a different set of primitive markers (Fig. 2C). In these comparisons, we also observed poor and/or even negative correlations, e.g., between primitive or cardiovascular genes and leukocyte markers, such as CD45 (Fig. 2D).

**Figure 3:**
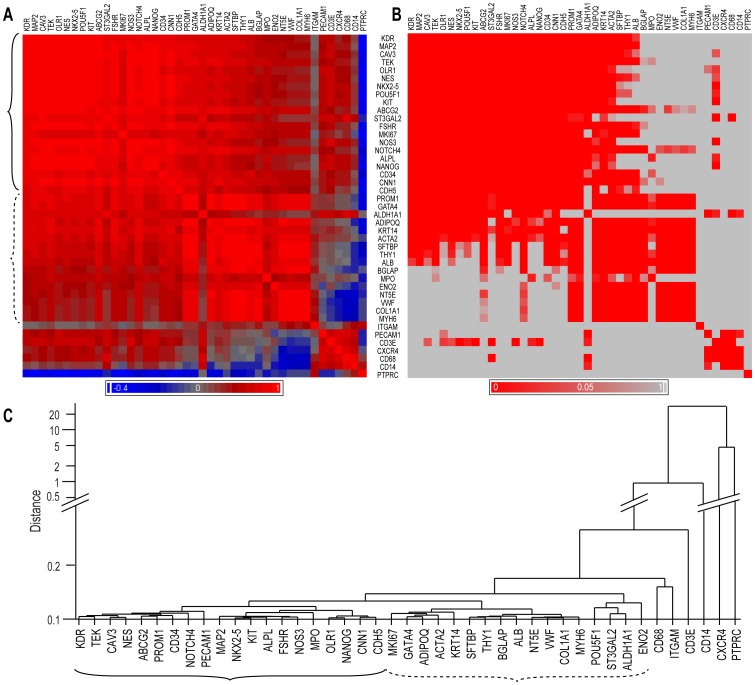
Correlation and clustering analysis of data. **A**. A heat map of the bivariate correlation matrix of gene expression levels representing Pearson's correlation coefficient, r, in descending order, beginning with KDR/VEGFR2 (red: positive correlation; blue: negative correlation; gray: no correlation). **B**. The corresponding probability values, p, after Bonferroni correction (red: p<0.05; gray: not significant). **C**. An unsupervised hierarchical clustering analysis (complete linkage on standardized data) representing the associations between the genes as distances (Y-axis). In **A** and **C**, the corresponding main gene groups are indicated by brackets.

These results were centralized in a covariation matrix (heat-map) using the hematopoietic/endothelial progenitor marker KDR/VEGFR2 as a reference [1] (Fig. 3A). The associated statistics matrix, after adjustment for multiple comparisons, revealed that a large majority of the Pearson coefficients were also statistically significant (Fig. 3B). The majority of primitive genes co-segregated with KDR in a group that contained most of the cardiovascular genes (Fig. 3A, B). This information was retrieved using unsupervised hierarchical clustering, thus objectively confirming that the majority of the primitive and cardiovascular genes aggregated together (Fig. 3C, solid bracket), while the leukocyte genes were less organized, and the tissue differentiation markers formed a separate cluster (Fig. 3C, dashed bracket).

### Network analysis of gene markers

**Figure 4:**
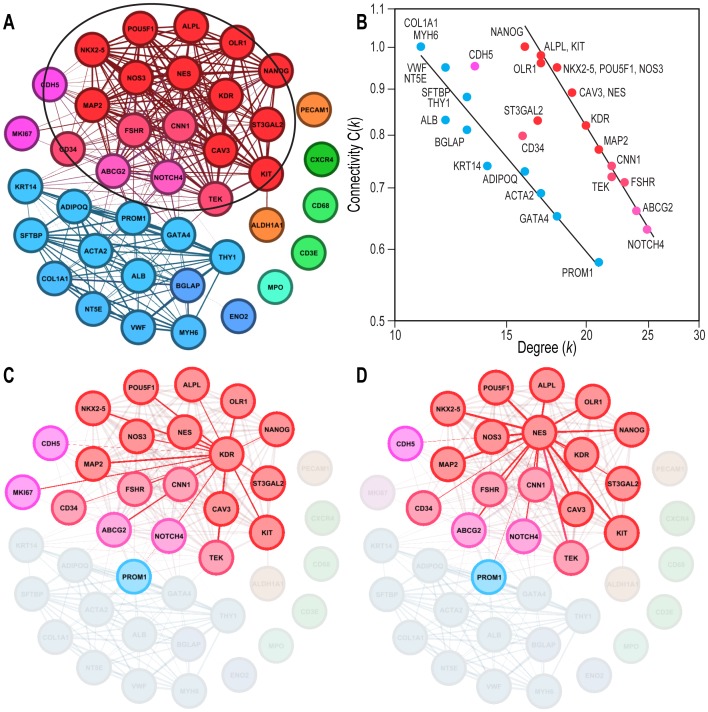
Modular organization of a PBMC gene sub-network. **A**. Network representation of the genetic covariation. The thickness of each connecting line is proportional to the absolute value of the respective Pearson's correlation coefficient. Genes that were significantly correlated with the age, AIx, AoPP and BMI of the subjects are encircled (cf. Table 3). Color coding identifies the participation of genes in separate underlying clusters [30]. **B**. Scaling of nodes' clustering coefficient C(*k*) with their connectivity degrees *k*, as a signature of hierarchical networks. Note that the data spontaneously split into two subpopulations, suggesting distinctly organized modules (for clarity, the leukocyte genes were omitted). Members of Module 1 (right), corresponding to the functionally filtered group in A (same color convention), had higher clustering values for similar *k* values than those in Module 2 (left), indicating stronger transcriptional coupling. **C**. Genes connected to the KDR node. Note that these connections perfectly overlap those of Module 1, while PROM1/CD133 serves as a link with Module 2, arguing that KDR is a hub node of Module 1. **D**. Connections of another hub node, NES. The images in **A, C** and **D** are based on Pearson correlation coefficients r>|0.8| and were obtained using the software Gephi 0.8 beta (www.gephi.org). The data shown in **B** were also obtained using Gephi, based on the network analysis in **A**.

Because the known CSPC types are characterized by multiple combinations of molecular markers [7], which were not well captured by dendrograms due to their linear character, we also used a network representation [34]. In this analysis, genes are considered nodes connected to other genes via links (or edges), based on a preset level of probabilistic significance (in our case, Pearson correlation coefficients larger than 0.8 at p<0.05). The number of edges between a node and the other genes to which it is connected is called its degree *k*, and the probability of its connections with all the other genes in the network is measured by a clustering coefficient C(*k*) [34]. This bottom-up analysis retrieved the grouping of marker genes shown in Fig. 3A as tightly interconnected gene communities [35] (Fig. 4A). To validate the modular properties of these communities, we displayed each node's clustering coefficient C(*k*) as dependent on its degree *k*. A negative relationship on a log-log scale between *k* and C(*k*) is considered the signature of hierarchical modularity in a network [34]. In this respect, our data objectively revealed the existence of two distinctly organized modules (Fig. 4B), each composed of the same assortment of primitive and differentiation markers as the clusters described in Fig. 3. The genes in Module 1 had higher values of clustering coefficients C(*k*) for any given value of their degree *k* compared with those in Module 2 (Fig. 4B). Some genes (e.g., CD34, ST3GAL2, and CDH5) had intermediate values of connectivity, indicating their position in a transition interface between the modules.

To further confirm the modular nature of this transcriptional network, some nodes (called hubs) were *directly* connected with all of the others in the corresponding module and with key nodes in the neighboring communities [34]. Based on this criterion, two nodes (KDR and NES, Fig. 4C, D) were connected with all of their neighbors, and both extended to PROM1/CD133 as a common contact node in Module 2, a property that prompted us to further investigate these nodes at the single-cell level (see below).

### Cardiovascular functional validation of genes from Module 1

To determine the physiological significance of the genes contributing to these co-expression patterns, we studied their individual relationships with the following characteristics of the blood donors: (i) age; (ii) the augmentation index (AIx) of their pulse, a surrogate measure of vascular stiffness [36]; (iii) blood pressure parameters (measured using applanation tonometry [37]); and (iv) body mass index (BMI). Significant correlations (p<0.05) were observed between these physiological variables and a group of 15 genes (Table 3), all of which belonged to the same module (Module 1, Fig. 3C, bracket; Fig. 4A, encircled; and Fig. 4B). These 15 genes were the following (as annotated in Table S1): (i) *primitive*: CD117/KIT, CD338/ABCG2, NANOG, NOTCH4, and POU5F1/OCT4; (ii) *primitive/endothelial*: CD34, KDR/VEGFR2, NES, NOS3/eNOS, OLR1/LOX-1, and TEK/TIE2, as well as ALPL (alkaline phosphatase, also mesenchymal), CNN1, and FSHR (neovascular); and (iii) *primitive/cardiac*: NKX2-5 and CAV3. The relationship between the Module 1 genes and BMI showed marked differences between the sexes; in females only, these genes were positively correlated with BMI. Additional Module 1 genes (and a few genes from Module 2) were significant in only two or three of the four tests (Table 2) and/or exhibited lower network connectivity at the interface of the two modules (Fig. 4B) and were thus not included in the definition of Module 1.

**Table 3:** Module 1 gene correlations with physiologic parameters.

Gene	Age		AIx(1)		AoPP(2)		BMI (females)(3)	
	r(4)	p-value	r	p-value	r	p-value	r	p-value
**ABCG2** (5)	**−0.463**	**0.0172**	**−0.430**	**0.0284**	**−0.447**	**0.0221**	**0.779**	**0.0006**
ADIPOQ	−0.469	0.0158	−0.364	0.0674	−0.306	0.1279	0.541	0.0371
**ALPL**	**−0.420**	**0.0327**	**−0.462**	**0.0176**	**−0.398**	**0.0442**	**0.889**	**0.0000**
**CAV3**	**−0.452**	**0.0203**	**−0.487**	**0.0116**	**−0.405**	**0.0403**	**0.828**	**0.0001**
CDH5	−0.461	0.0177	−0.340	0.0892	−0.348	0.0819	0.830	0.0001
**CNN1**	**−0.475**	**0.0142**	**−0.490**	**0.0110**	**−0.469**	**0.0157**	**0.776**	**0.0007**
**FSHR**	**−0.514**	**0.0073**	**−0.507**	**0.0083**	**−0.476**	**0.0139**	**0.772**	**0.0007**
GATA4	−0.446	0.0223	−0.410	0.0355	−0.286	0.1566	0.597	0.0187
**KDR**	**−0.398**	**0.0442**	**−0.420**	**0.0324**	**−0.399**	**0.0481**	**0.830**	**0.0001**
**KIT**	**−0.451**	**0.0209**	**−0.470**	**0.0155**	**−0.414**	**0.0356**	**0.853**	**0.0001**
KRT14	−0.436	0.0258	−0.425	0.0306	−0.416	0.0348	0.129	0.5301
MAP2	−0.512	0.0074	−0.470	0.0155	−0.341	0.0886	0.834	0.0001
MKI67	−0.479	0.0132	−0.375	0.0592	−0.232	0.2548	0.773	0.0007
**NANOG**	**−0.468**	**0.0159**	**−0.464**	**0.0169**	**−0.451**	**0.0209**	**0.833**	**0.0001**
**NES**	**−0.519**	**0.0065**	**−0.507**	**0.0083**	**−0.413**	**0.0359**	**0.832**	**0.0001**
**NKX2-5**	**−0.462**	**0.0176**	**−0.469**	**0.0155**	**−0.410**	**0.0374**	**0.845**	**0.0001**
**NOS3**	**−0.460**	**0.0180**	**−0.527**	**0.0057**	**−0.520**	**0.0065**	**0.764**	**0.0009**
**NOTCH4**	**−0.457**	**0.0190**	**−0.494**	**0.0103**	**−0.458**	**0.0187**	**0.738**	**0.0017**
**OLR1**	**−0.526**	**0.0058**	**−0.479**	**0.0133**	**−0.406**	**0.0396**	**0.823**	**0.0002**
**POU5F1**	**−0.493**	**0.0104**	**−0.478**	**0.0136**	**−0.462**	**0.0175**	**0.824**	**0.0002**
PROM1	−0.489	0.0112	−0.398	0.0438	−0.259	0.2012	0.563	0.0288
**TEK**	**−0.480**	**0.0132**	**−0.471**	**0.0152**	**−0.412**	**0.0366**	**0.766**	**0.0009**

(1) Augmentation index, a surrogate measure of vascular stiffness extracted from radial pulse wave measurements, as described in the Methods.

(2) Aortic pulse pressure.

(3) Significant correlations with BMI were found only in females.

(4) Pearson's correlation coefficient.

(5) Genes that were significantly correlated with all four parameters are in bold.

### Personalized representation of gene analysis

**Figure 5:**
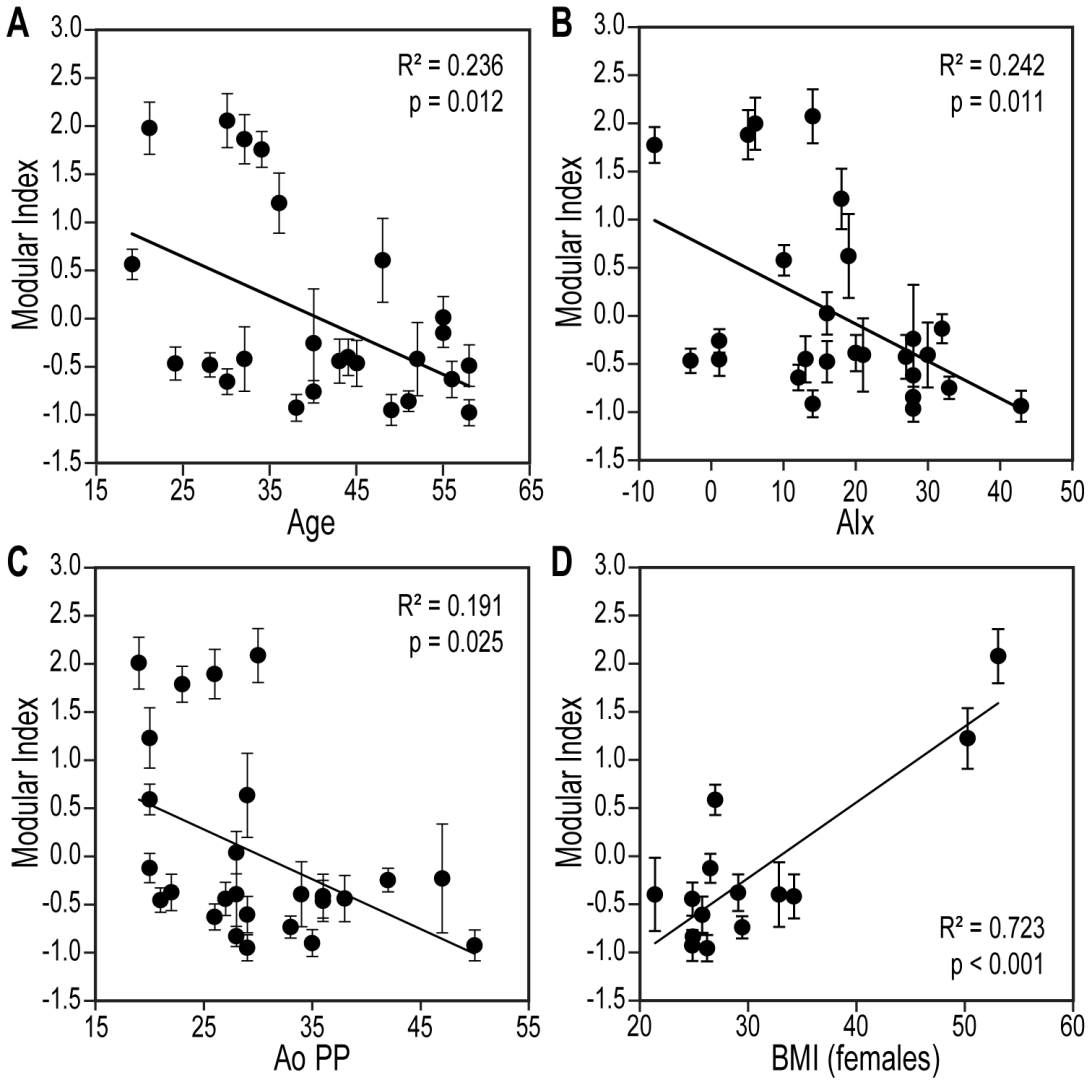
Modular index (MI) of Module 1 (metagene) associated with the age and cardiovascular parameters of blood donors. **A**. Age-dependent variation in MI in the population. **B**. Regression analysis of MI on AIx. **C, D**. Correlations of MI with AoPP and BMI (in women). MI represents the 15-gene average of the standardized (mean = 0, SD = 1) RCN of each gene within the tested population, +/− SD. n = 26 for **A–C** and n = 14 for **D**. Note the apparently bimodal distribution of MI with age in this population.

We sought to consolidate the individual gene expression information into a parameter that would combine the contributions of all of the 15 Module 1 genes that exhibited significance in the above correlations with the physiological variables (Table 2). For each healthy subject, we calculated a modular index (MI) (also known as a metagene) [17], the average of the standardized RCN values of the module's genes (Fig. 5). As expected from the dependence of individual genes on the respective physiological variables, the MI values of all the subjects exhibited negative correlations with age (Fig. 5A), vascular stiffness (Fig. 5B), and aortic pulse pressure (Fig. 5C), as well as a positive correlation with BMI in females (Fig. 5D).

Additionally, we investigated whether these 15 genes were *sufficient* for the ability of MI to correctly characterize the correlation between the metagene and the respective physiological variable (e.g., age, AIx, and blood pressure). Therefore, we calculated the Cronbach's alpha coefficient for internal consistency, defined as the correlation between different test items (in our case, the genes), that determines whether these items collectively represent the same general construct [38]. High Cronbach's alpha coefficients are generally desirable, but if too high (conventionally >0.95), they may indicate item redundancy [38]. For the genes contained in the metagene MI, the Cronbach's coefficient was indeed >0.95, indicating that this set of variables was not only consistent but also saturated.

**Figure 6:**
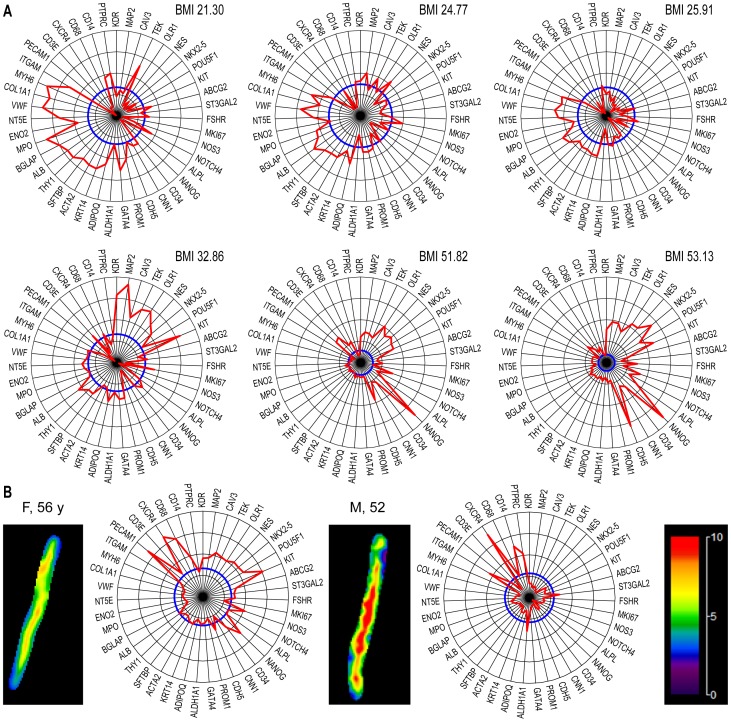
Personalized representation of gene expression as radial graphs. **A**. The reference population level (RCN median, n = 26, 100%) is shown in blue, and the corresponding individual percentage values for the specified genes are shown in red in the order used in Fig. 2A. Note the pattern differences in Module 1 genes between females with normal BMIs (upper row) and those with higher BMIs (lower row). **B**. Association of aortic stiffness (left-side images) with radial gene profiles (right-side graphs) of two subjects: *left*, female, 56 years, average stiffness of 5.25 kPa; right: male, 52 years, average stiffness of 6.17 kPa. Aortic stiffness was measured by MRE, as described the Methods section; the local elasticity distribution is color coded, as shown in the scale at right (in kPa).

For a more detailed representation of gene-specific information, we also generated radial diagrams that displayed the individual values of analyzed genes as the percentages vs. the median levels in the population. For example, we compared the personalized diagrams of female blood donors with normal weights against those with BMIs in the overweight range (Fig. 6A). We noted that the sector of the diagram containing Module 1 genes was better represented in the latter group (in agreement with the positive correlation with BMI found for Module 1 genes in women, Fig. 5D), while the relative amplitude of Module 2 members decreased progressively.

The benefit of this representation was also illustrated by comparing gene expression patterns in subjects with various degrees of vascular stiffness, objectively determined by MRE. The results showed a reduction of gene expression in both modules with increasing aortic stiffness in these otherwise healthy subjects (Fig. 6B).

### Modifications of PBMC gene expression in hypertensive patients

**Figure 7:**
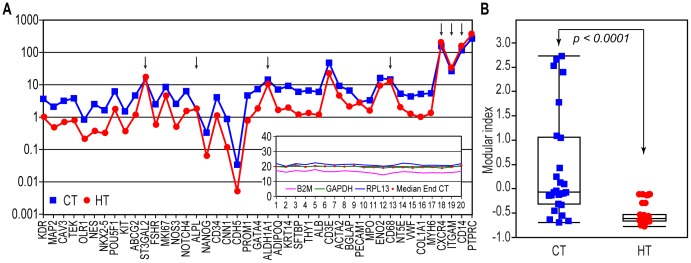
Comparison of gene expression in healthy and hypertensive subjects. **A**. The median RCN in healthy controls (CT, blue) vs. hypertensive patients (HT, red); all of the differences are significant (p<0.05), with the exception of those labeled by arrows. *Inset*. Cq values of housekeeping genes in this patient population. **B**. Box plots of the aggregated MI (averages of normalized RCN values of Module 1 genes) in the control subjects and patients. (Box plots show the median values, 1^st^ and 3^rd^ quartiles, and the interquartile range; symbols are as in **A**).

**Figure 8:**
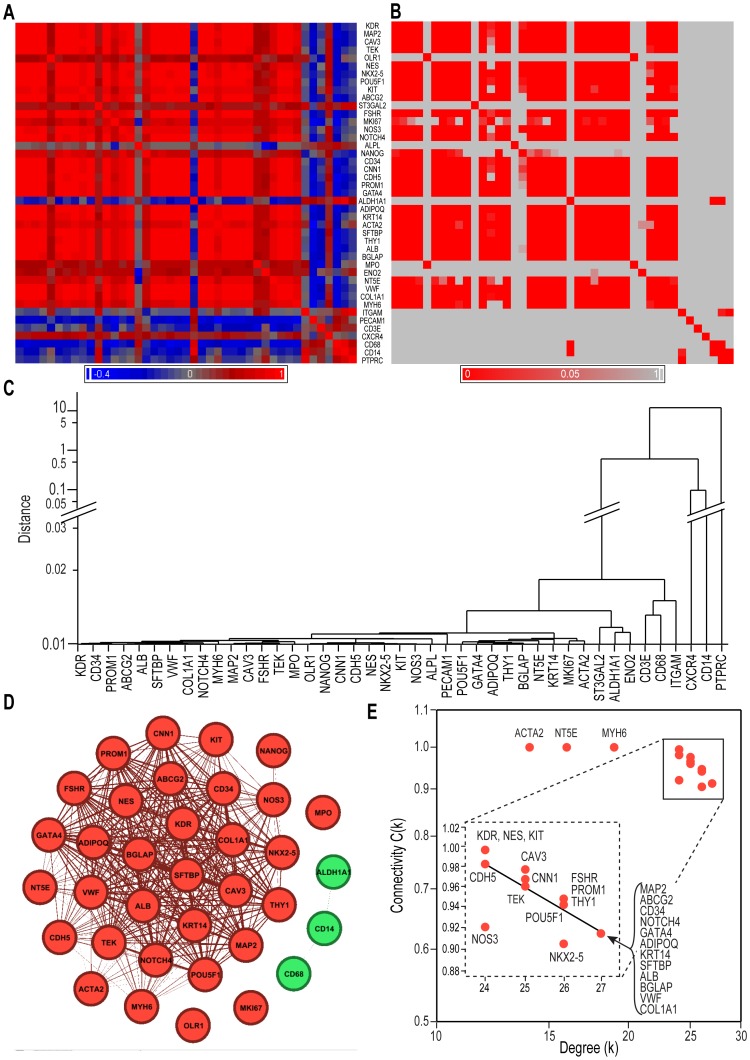
Changes in the modular organization of genes in hypertensive patients (n = 20; see Figs. 3 and 4 for details). **A**. A heat map of intergenic covariation. **B**. The corresponding matrix of significance values following Bonferroni corrections. **C**. Dendrogram of hierarchical gene clustering. **D**. The network structure of the patient genes, indicating the fusion of Modules 1 and 2 of the network found in the healthy subjects (red). **E**. The relationship between the gene clustering coefficient C(*k*) and node degree (connectivity) *k*; the collapsed sub-network shows a very strong and nearly uniform connection between nodes (inset), suggestive of transcriptional primitivity. The data analysis and representation were performed as in Figs. 3 and 4.

The genes from Module 1 had inverse correlations with several cardiovascular risk factors (age, blood pressure, and aortic stiffness), which suggested that even stronger alterations might be found in patients with established vascular pathologies. To test this hypothesis, we utilized a cohort of cardiovascular patients treated for hypertension in an outpatient cardiovascular clinic (Table 3). We found that the expression levels of the tested genes in PBMCs from these patients closely paralleled those in healthy subjects (Fig. 7A). However, with a few exceptions involving non-significant differences (Fig. 7A), the RCNs were reduced compared with the healthy population, an observation also captured using their metagene MI (Fig. 7B). Covariation heat-maps revealed obvious disruptions in the co-expression patterns of these genes in the patients compared with the healthy controls (Fig. 8A, B; compare with Fig. 3A, B). The hierarchical clustering was also sensitive to these rearrangements (Fig. 8C). The network representation showed that the connectivity of nodes within and between both modules was largely increased (Fig. 8D), as they collapsed in a common, highly coupled sub-network (Fig. 8E). Remarkably, the *range* of connectivity degree *k* values of these module's nodes in patients was reduced (23–26, Fig. 8E) compared with that in the healthy subjects (17–25, Fig. 4B), arguing for lower connection variability among node genes.

**Figure 9:**
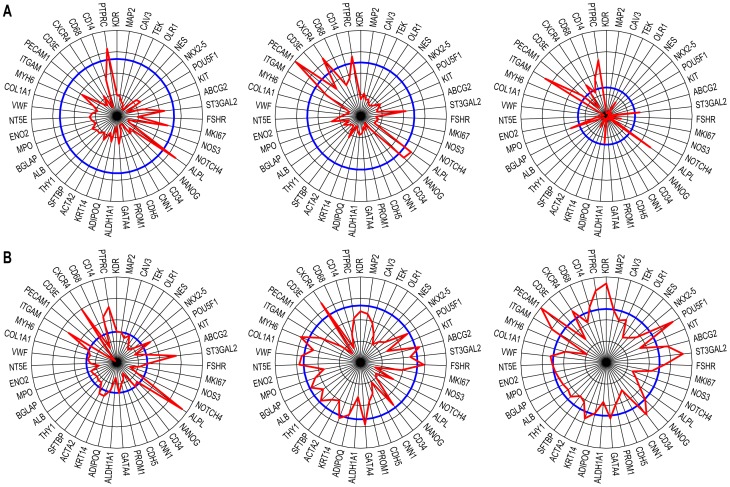
Radial representation of relative gene expression in hypertensive patients. **A**. Representative radial diagrams of patients with hypertension. Note the overall reduction in expression, with the exception of a subgroup of genes (e.g., ALPL, ITGAM, and PTPRC/CD45; compare with Fig. 7A). **B**. Radial diagrams of hypertensive patients treated with thiazide; in these cases, the gene patterns in the two modules were closer to normal (i.e. closer to the blue reference line).

The radial diagrams indicated that in addition to the conspicuous reduction in expression levels in both modules (Fig. 9A), the patients treated with the diuretic thiazide displayed gene levels closer to normal than those taking other medications (Fig. 9B). The patients with hypertension exhibited unchanged levels of ALPL, a tissue-type alkaline phosphatase gene expressed by many non-vascular cells (including osteoblasts [39]) but also by vascular lineage CSPCs [40]. The following genes also exhibited module-independent variations among patients: (i) ALDH1A1, a marker of primitivity with numerous cardiovascular implications [41]; (ii) CXCR4, the receptor for SDF-1, which is essential for CSPC recruitment [42]; and (iii) ST3GAL2, a transcriptional marker of SSEA-4 [43], which has been associated with adult bone marrow-derived mesenchymal stem cells [44], and with the controversial [45] very small embryonic-like stem cells [46].

### Cellular origins of gene co-expression

**Figure 10:**
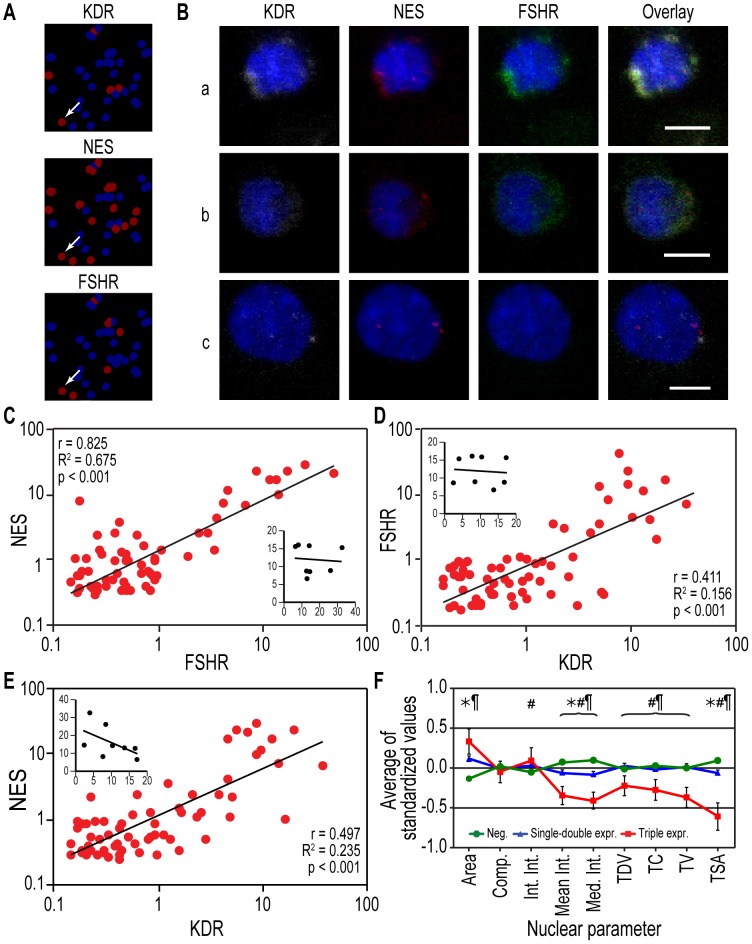
Origins of marker gene co-expression within individual cells: *in situ* hybridization (ISH). **A**. ISH analysis of the Module 1 hub genes KDR and NES and the node gene FSHR, identified by their fluorescent signals in a given microscopic field (brown masks were added to positive cells by the CellProfiler image analysis software; blue represents DAPI staining of nuclei). *Arrow*: a triple-positive cell. **B**. Four-color confocal images of cells that are positive for (**a**) all three markers; (**b**) NES and FSHR only; or (**c**) KDR and NES only (white: KDR; red: NES; green: FSHR; blue: nuclei). Bars: 5 µm. **C–E**. Linear regression of the integrated pixel intensity of the mRNA of each marker gene (KDR, NES, and FSHR) detected using ISH in triple-positive cells (n = 66 cells pooled from 8 individuals; r: Pearson's correlation coefficient; r^2^: regression coefficient; log-log scale). Inset graphs show the lack of correlation between mRNA expression (also measured as the integrated pixel intensity) in single-positive cells for each respective pair of markers. **F**. Nuclear area and several texture features calculated using the CellProfiler analysis significantly separated the triple-positive cells from the other cells (*, p<0.05 for single and double expressers vs. negatives; #, p<0.05 for single and double expressers vs. triple positives; ¶, p<0.05 for triple expressers vs. negatives) (a total of 2094 cells from 8 subjects were analyzed). Abbreviations: Comp., compactness; Int. Int., integrated intensity; Mean Int., mean intensity; Med. Int., median intensity; TDV, texture difference variance; TC, texture contrast; TV, texture variance; TSA, texture sum average. The data represent the means of standardized values ± SEM.

To identify the origins of gene expression we analyzed mRNA and protein expression at the single-cell level. We used cytospun PBMCs probed for the Module 1 hub nodes KDR and NES. To these, we added the follicle-stimulating hormone receptor (FSHR), a marker of neovascular endothelium [47], which was strongly correlated with the other two nodes (Fig. 2A and Table S1). For mRNA detection, we performed *in situ* hybridization, followed by an automated image analysis. In all of the tested samples, we found multiple cells expressing these marker mRNAs in various proportions (Fig. 10A, B). To determine whether the Module 1 covariation pattern was derived from the coordinated presence in the circulation of cells *separately* expressing these genes, we performed two-by-two comparisons of the frequencies of single-positive cells in the same individuals, which did not show a correlation (Fig. 10C–E, insets). Instead, in support of a genuine within-cell co-expression mechanism, we found a high proportionality of the signals from all three mRNAs only within triple-positive cells (Fig. 10C–E).

Given that nuclear morphology is sensitive to a cell's transcriptional activity [48], we examined whether nuclear parameters obtained by image analysis could distinguish the cells co-expressing the Module 1-related mRNAs form the other PBMCs. Indeed, nuclear shape and texture analysis showed that the nuclei of cells triple-positive for KDR/NES/FSHR exhibited a distinct morphology (Fig. 10F).

**Figure 11:**
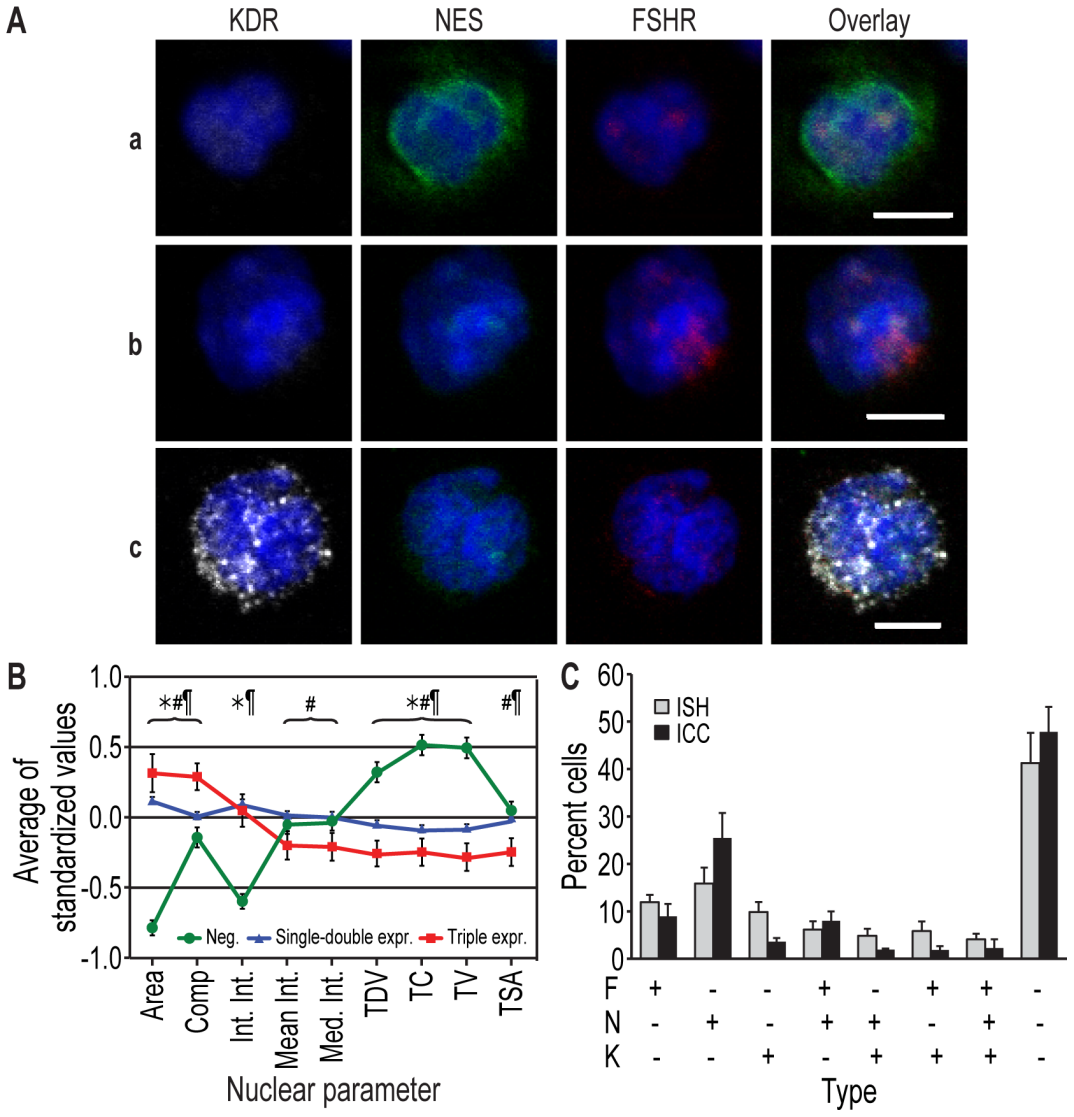
Detection of cells expressing representative Module 1 node proteins using immunocytochemistry (ICC). **A**. Cells expressing various levels of KDR (white), NES (green), and/or FSHR (red). Nuclei are blue (DAPI). **a**, **b**: NES-FSHR double-positive cells; **c**: a triple-positive cell. Bars: 5 µm. **B**. A nuclear morphology analysis revealed alterations in the triple-positive cells detected using ICC that were comparable to those found using ISH (see Fig. 10 for abbreviations). The data represent the means of standardized values ± SEM; a total of 1655 cells were analyzed. **C**. Frequencies of cells positive for the three marker genes, detected using ISH (gray bars) and ICC (black bars) (n = 2094 and 1655 cells, respectively; none of the inter-method comparisons were significant, demonstrating that they detected the same cell populations). The data represent the means of individual blood donors ± SEM.

Finally, we searched for the proteins encoded by the mRNAs of interest by performing immunocytochemistry on parallel slides and found cells expressing all possible combinations of these antigens (Fig. 11A). The nuclear morphology of the triple-positive cells revealed by immunocytochemistry was similar to that detected by *in situ* hybridization, i.e., a larger apparent nuclear area and less textured compared with the other cells (Fig. 11B), thus confirming that the two methods detected the same cellular populations. In support of this observation, the frequency distributions of the antigen expression classes (i.e., single, double, or triple expressers) were also similar to those detected using mRNA *in situ* hybridization in blood samples from the same individuals (Fig. 11C).

## Discussion

Here, we report several novel observations derived from an analysis of transcriptional activity in PBMCs: (i) unlike GeneChips, which failed to reliably detect approximately half of the gene targets, qRT-PCR detected all 45 members of a panel of primitive and differentiation marker genes in all the tested human blood samples; (ii) based on their strong covariation, the target genes segregated into two major clusters, which exhibited the connectivity properties of modules in a bottom-up reconstituted hierarchical transcriptional network; (iii) one of modules contained most of the primitive and cardiovascular differentiation markers; (iv) this module also correlated with several cardiovascular risk factors in the healthy blood donors, contributing to a cardiovascular-specific metagene; (v) the origin of genetic covariation was found within individual cells of a subpopulation with distinctive nuclear properties; (vi) the levels of gene expression in both modules were significantly reduced in hypertensive patients; and (vii) the connectivity within the PBMC gene modules was largely amplified in hypertensive patients, leading to the fusion of these modules into a common sub-network.

The properties of these gene modules were consistent with what would be expected if they derived from CSPCs; in particular, Module 1 could be the signature of circulating endothelial progenitor cells. The dependence of Module 1 genes on BMI (in women) is in agreement with the finding that CSPCs are under the influence of adipose tissue, both as a source of pro-angiogenic chemokines (e.g., adiponectin, known to induce the release of CSPCs from bone marrow [49]) and as a possible direct originator of CSPCs [50]. However, these gene modules were not equated with any known underlying cellular classes for the following reasons: (i) the stochastic nature of gene expression [51], [52]; (ii) cell plasticity, manifested as a transcriptionally dependent propensity for transdifferentiation [53]; and (iii) the possible horizontal redistribution of mRNA among different cell classes, possibly via extracellular vesicles acting as intercellular RNA carriers [54], which are particularly active among bone marrow stem cells [55]. Instead, we consider that although the Module 1 markers are primarily expressed by EPCs [7], they also receive contributions from other leukocytes with roles in maintaining vascular function, such as angiogenic (TEK/TIE2^+^) monocytes [56] or angiogenic T cells [57]. Other circulating bone marrow-derived cells of less certain nature also contribute to the maintenance of microvascular tone and normal blood pressure [58].

Equally important, many T lymphocytes positive for CD31 and CXCR4 were found at the core of the *in vitro*-formed colonies known as ‘early’ EPCs [59], which are bordered by KDR/VEGFR2^+^ monocytic cells [60]. Despite their obvious non-endothelial nature, the numbers of these heterogeneous, blood-derived cell aggregates were highly correlated with the vascular function of the blood donors [61]. In a larger context, leukocytes often express other tissue-specific genes, revealing a refined but poorly understood transcriptional cross-talk between blood and perfused tissues [15]. Thus, we consider that the gene expression pattern detected using our method diverges from the simple notion of circulating progenitor cells into a more complex underlying biological reality that is highly meaningful for vascular function. In addition, we assume that the Module 1 genes do not derive from circulating adult endothelial cells detached from the vascular intima, because the endothelial functional marker von Willebrand factor was not present among these genes. The fact that a class of cells with distinctive nuclear properties, suggestive of a transcriptionally active euchromatin, coordinately express the neovascular markers KDR, NES and FSHR, provides additional evidence that Module 1 genes derive from primitive rather than differentiated cells. However, we consider highly unlikely that all the 15 members of Module 1 to be simultaneously expressed by only one cell category, and thus we maintain that the origin of Module 1 is an emergent property of the CSPCs system.

Vascular stiffness determines an individual's susceptibility to atherosclerotic plaque formation by predisposing the intimal endothelium to increased permeability to lipoproteins and the accumulation of monocytes [62]. Therefore, the presence of endothelial differentiation genes associated with primitive genes in the structure of Module 1 and their inverse correlation with AIx indicate a protective role of the respective blood cells in vessel health. Of note, AIx did not exhibit a direct relationship with the age of the blood donors in our limited population, arguing that the dependence of AIx on Module 1 components is not *indirectly* mediated through the effect of age on the expression of these components. The decrease in the levels of gene expression in PBMCs with an increase in the blood donor's age may be due to variations in the number of gene-expressing cells, changes in these cells' transcriptional activity (such as the known aging-sensitive dependence of transcription on the methylation of CpG islands in gene regulatory elements [63]), or both. Additionally, the inverse association between Module 1-derived MI and central aortic pulse pressure (AoPP), which was recently shown to predict the future development of hypertension in healthy human populations [64], suggests a potential role of Module 1 genes in protecting small resistance vessels.

Decreased collective expression of the genes in both modules was observed among the patients with established (and treated) hypertension. This finding is consistent with the reduction of CSPCs, including CD34-positive cells [65], in patients with hypertension, possibly as a direct effect of angiotensin [66] or in response to anti-hypertensive treatments [67]. Other novel observations of this study are that in the hypertensive patients, there is increased *covariation* of the genes in both modules and the distinction between these two modules vanishes. The increased network connectivity in the patients, despite the overall reduction in expression levels, suggests an amplified transcriptional coordination. In terms of gene network organization, the observed reduced informational heterogeneity is the signature of a less-differentiated state of the marker's originating cells, which is consistent with the higher transcriptional network entropy of primitive cells [68]. In support of this possibility, hematopoiesis is amplified in cardiovascular patients, based on blood gene expression signatures [17].

Among the limitations of this study is the relatively small population sample size. Despite this limitation, the correlations between genes were strong and significant, highlighting the power of our bottom-up network reconstruction method to extract meaningful information from small human populations. Admittedly, the set of genes examined here could still be an incomplete representation of the actual underlying module from where our target markers were extracted. This topic is worthy of future exploration, although we demonstrated that the number of components was sufficient for the use of Module 1 in the current form as an aggregate biomarker (or metagene) of vascular function. Finally, the other module that surfaced from our analysis (Module 2 in normal subjects) remains to be explored to determine its significance for the repair capacity of blood, which may occur in an organ- and/or disease-specific manner.

In conclusion, the results reported herein constitute the proof of concept for a novel bottom-up approach that is more sensitive and more accurate than the currently used high-throughput methods for the generation of a peripheral blood transcriptional network module useful for studying the collective contribution of circulating cells to vascular and tissue maintenance and repair.

## Supporting Information

Table S1 Abbreviations: Angio: angiogenesis; EC: endothelial cells; EPC: endothelial progenitor cells; FB: fibroblasts; MC: monocytes; Mph: macrophages; MSC: mesenchymal stem cells; SMC: smooth muscle cells; VSELC: very small embryonic-like stem cells. For some genes, alternative frequently used names are given in parentheses.
(DOCX)

Table S2 ^(1)^ In those instances when a gene was represented by several probe sets on the array, we calculated the median signal value for each probe set over all of the arrays and retained the sets that had the highest value. ^(2)^ Presence Score was calculated as percentage from all 274 arrays, using MAS5 algorithm (Affymetrix Expression Console). The following GEO datasets were used to generate these data: GSE8507, GSE10041, GSE11761, GSE14642, GSE19743, GSE21942, GSE27034, and GSE46480 ^(3)^ Percentages in this column are calculated only from the arrays used in this study [1]. Reference: 1 Li L, Li M, Sun C, Francisco L, Chakraborty S, et al. (2011) Altered hematopoietic cell gene expression precedes development of therapy-related myelodysplasia/acute myeloid leukemia and identifies patients at risk. Cancer Cell 20: 591–605. (GSE23025).
(DOCX)

## References

[1] Asahara T, Murohara T, Sullivan A, Silver M, van der Zee R, et al (1997) Isolation of putative progenitor endothelial cells for angiogenesis. Science 275: 964–967. 9020076 10.1126/science.275.5302.964

[2] Li CS, Neu MB, Shaw LC, Kielczewski JL, Moldovan NI, et al (2010) EPCs and pathological angiogenesis: when good cells go bad. Microvasc Res 79: 207–216. 20188747 10.1016/j.mvr.2010.02.011 PMC3650470

[3] Yang Z, von Ballmoos MW, Faessler D, Voelzmann J, Ortmann J, et al (2010) Paracrine factors secreted by endothelial progenitor cells prevent oxidative stress-induced apoptosis of mature endothelial cells. Atherosclerosis 211: 103–109. 20227693 10.1016/j.atherosclerosis.2010.02.022

[4] Obokata H, Wakayama T, Sasai Y, Kojima K, Vacanti MP, et al (2014) Stimulus-triggered fate conversion of somatic cells into pluripotency. Nature 505: 641–647. 24476887 10.1038/nature12968

[5] Dong C, Goldschmidt-Clermont PJ (2007) Endothelial progenitor cells: a promising therapeutic alternative for cardiovascular disease. J Interv Cardiol 20: 93–99. 17391216 10.1111/j.1540-8183.2007.00251.x

[6] Palombo C, Kozakova M, Morizzo C, Gnesi L, Barsotti MC, et al (2011) Circulating endothelial progenitor cells and large artery structure and function in young subjects with uncomplicated type 1 diabetes. Cardiovasc Diabetol 10: 88 10.1186/1475-2840-10-88 21981808 PMC3198903

[7] Richardson MR, Yoder MC (2011) Endothelial progenitor cells: quo vadis? J Mol Cell Cardiol 50: 266–272. 20673769 10.1016/j.yjmcc.2010.07.009 PMC3444239

[8] Duda DG, Cohen KS, Scadden DT, Jain RK (2007) A protocol for phenotypic detection and enumeration of circulating endothelial cells and circulating progenitor cells in human blood. Nat Protoc 2: 805–810. 17446880 10.1038/nprot.2007.111 PMC2686125

[9] Hill JM, Zalos G, Halcox JP, Schenke WH, Waclawiw MA, et al (2003) Circulating endothelial progenitor cells, vascular function, and cardiovascular risk. N Engl J Med 348: 593–600. 12584367 10.1056/NEJMoa022287

[10] Tura O, Skinner EM, Barclay GR, Samuel K, Gallagher RC, et al (2013) Late outgrowth endothelial cells resemble mature endothelial cells and are not derived from bone marrow. Stem Cells 31: 338–348. 23165527 10.1002/stem.1280

[11] Barabasi AL, Gulbahce N, Loscalzo J (2011) Network medicine: a network-based approach to human disease. Nat Rev Genet 12: 56–68. 21164525 10.1038/nrg2918 PMC3140052

[12] Palmer NP, Schmid PR, Berger B, Kohane IS (2012) A gene expression profile of stem cell pluripotentiality and differentiation is conserved across diverse solid and hematopoietic cancers. Genome Biol 13: R71–R84. 22909066 10.1186/gb-2012-13-8-r71 PMC3491371

[13] Wojakowski W, Tendera M, Kucia M, Zuba-Surma E, Paczkowska E, et al (2009) Mobilization of bone marrow-derived Oct-4+ SSEA-4+ very small embryonic-like stem cells in patients with acute myocardial infarction. J Am Coll Cardiol 53: 1–9. 19118716 10.1016/j.jacc.2008.09.029 PMC5536894

[14] Liew CC, Ma J, Tang HC, Zheng R, Dempsey AA (2006) The peripheral blood transcriptome dynamically reflects system wide biology: a potential diagnostic tool. J Lab Clin Med 147: 126–132. 16503242 10.1016/j.lab.2005.10.005

[15] Kohane IS, Valtchinov VI (2012) Quantifying the white blood cell transcriptome as an accessible window to the multiorgan transcriptome. Bioinformatics 28: 538–545. 22219206 10.1093/bioinformatics/btr713 PMC3288749

[16] de JS, Boks MP, Fuller TF, Strengman E, Janson E, et al (2012) A gene co-expression network in whole blood of schizophrenia patients is independent of antipsychotic-use and enriched for brain-expressed genes. PLoS One 7: e39498 10.1371/journal.pone.0039498 22761806 PMC3384650

[17] Joehanes R, Ying S, Huan T, Johnson AD, Raghavachari N, et al (2013) Gene expression signatures of coronary heart disease. Arterioscler Thromb Vasc Biol 33: 1418–1426. 23539218 10.1161/ATVBAHA.112.301169 PMC3684247

[18] Kang BH, Jensen KJ, Hatch JA, Janes KA (2013) Simultaneous profiling of 194 distinct receptor transcripts in human cells. Sci Signal 6: rs13 10.1126/scisignal.2003624 23921087 PMC3772518

[19] Alberts R, Lu L, Williams RW, Schughart K (2011) Genome-wide analysis of the mouse lung transcriptome reveals novel molecular gene interaction networks and cell-specific expression signatures. Respir Res 12: 61. 21535883 10.1186/1465-9921-12-61 PMC3105947

[20] Bock C, Kiskinis E, Verstappen G, Gu H, Boulting G, et al (2011) Reference Maps of human ES and iPS cell variation enable high-throughput characterization of pluripotent cell lines. Cell 144: 439–452. 21295703 10.1016/j.cell.2010.12.032 PMC3063454

[21] Guo G, Luc S, Marco E, Lin TW, Peng C, et al (2013) Mapping Cellular Hierarchy by Single-Cell Analysis of the Cell Surface Repertoire. Cell Stem Cell 13: 492–505. 24035353 10.1016/j.stem.2013.07.017 PMC3845089

[22] Moignard V, Macaulay IC, Swiers G, Buettner F, Schutte J, et al (2013) Characterization of transcriptional networks in blood stem and progenitor cells using high-throughput single-cell gene expression analysis. Nat Cell Biol 15: 363–372. 23524953 10.1038/ncb2709 PMC3796878

[23] Kucia M, Zhang YP, Reca R, Wysoczynski M, Machalinski B, et al (2006) Cells enriched in markers of neural tissue-committed stem cells reside in the bone marrow and are mobilized into the peripheral blood following stroke. Leukemia 20: 18–28. 16270036 10.1038/sj.leu.2404011

[24] AtCorMedical (2013) A clinical guide: Pulse wave analysis. http://www.atcormedical.com/pdf/Manuals/SphygmoCor%20Software%20Guide%20Px.pdf. Accessed 2014 March 30.

[25] Kolipaka A, Woodrum D, Araoz PA, Ehman RL (2012) MR elastography of the in vivo abdominal aorta: a feasibility study for comparing aortic stiffness between hypertensives and normotensives. J Magn Reson Imaging 35: 582–586. 22045617 10.1002/jmri.22866 PMC3401065

[26] Damughatla AR, Raterman B, Sharkey-Toppen T, Jin N, Simonetti OP, et al. (2013) Quantification of aortic stiffness using MR Elastography and its comparison to MRI-based pulse wave velocity. J Magn Reson Imaging. doi:10.1002/jmri.24506. PMC4318508 24243654

[27] Gavrilin MA, Bouakl IJ, Knatz NL, Duncan MD, Hall MW, et al (2006) Internalization and phagosome escape required for Francisella to induce human monocyte IL-1beta processing and release. Proc Natl Acad Sci U S A 103: 141–146. 16373510 10.1073/pnas.0504271103 PMC1324976

[28] Carpenter AE, Jones TR, Lamprecht MR, Clarke C, Kang IH, et al (2006) CellProfiler: image analysis software for identifying and quantifying cell phenotypes. Genome Biol 7: R100. 17076895 10.1186/gb-2006-7-10-r100 PMC1794559

[29] Li L, Li M, Sun C, Francisco L, Chakraborty S, et al (2011) Altered hematopoietic cell gene expression precedes development of therapy-related myelodysplasia/acute myeloid leukemia and identifies patients at risk. Cancer Cell 20: 591–605. 22094254 10.1016/j.ccr.2011.09.011 PMC3220884

[30] Xiang Y, Fuhry D, Kaya K, Jin R, Catalyurek UV, et al (2012) Merging network patterns: a general framework to summarize biomedical network data. Netw Model Anal Health Inform Bioinforma 1: 103–116.

[31] Bron C (1973) Algorithm 457: finding all cliques of an undirected graph. Commun ACM 16: 575–577.

[32] Bastian M, Heymann S, Jacomy M (2009) Gephi: An open source software for exploring and manipulating networks. International AAAI Conference on Weblogs and Social Media.

[33] Etienne W, Meyer MH, Peppers J, Meyer RA Jr (2004) Comparison of mRNA gene expression by RT-PCR and DNA microarray. Biotechniques 36: 618–6. 15088380 10.2144/04364ST02

[34] Barabasi AL, Oltvai ZN (2004) Network biology: understanding the cell's functional organization. Nat Rev Genet 5: 101–113. 14735121 10.1038/nrg1272

[35] West J, Widschwendter M, Teschendorff AE (2013) Distinctive topology of age-associated epigenetic drift in the human interactome. Proc Natl Acad Sci U S A 110: 14138–14143. 23940324 10.1073/pnas.1307242110 PMC3761591

[36] Riggio S, Mandraffino G, Sardo MA, Iudicello R, Camarda N, et al (2010) Pulse wave velocity and augmentation index, but not intima-media thickness, are early indicators of vascular damage in hypercholesterolemic children. Eur J Clin Invest 40: 250–257. 20415700 10.1111/j.1365-2362.2010.02260.x

[37] Garcia-Ortiz L, Recio-Rodriguez JI, Canales-Reina JJ, Cabrejas-Sanchez A, Gomez-Arranz A, et al (2012) Comparison of two measuring instruments, B-pro and SphygmoCor system as reference, to evaluate central systolic blood pressure and radial augmentation index. Hypertens Res 35: 617–640. 22297480 10.1038/hr.2012.3

[38] Streiner DL (2003) Starting at the beginning: an introduction to coefficient alpha and internal consistency. J Pers Assess 80: 99–103. 12584072 10.1207/S15327752JPA8001_18

[39] Heinemann DE, Siggelkow H, Ponce LM, Viereck V, Wiese KG, et al (2000) Alkaline phosphatase expression during monocyte differentiation. Overlapping markers as a link between monocytic cells, dendritic cells, osteoclasts and osteoblasts. Immunobiology 202: 68–81. 10879691 10.1016/S0171-2985(00)80054-6

[40] Fadini GP, Albiero M, Menegazzo L, Boscaro E, Agostini C, et al (2012) Procalcific phenotypic drift of circulating progenitor cells in type 2 diabetes with coronary artery disease. Exp Diabetes Res 2012: 921685 10.1155/2012/921685 22474430 PMC3299316

[41] Balber AE (2011) Concise review: aldehyde dehydrogenase bright stem and progenitor cell populations from normal tissues: characteristics, activities, and emerging uses in regenerative medicine. Stem Cells 29: 570–575. 21308868 10.1002/stem.613

[42] Penn MS (2010) SDF-1:CXCR4 axis is fundamental for tissue preservation and repair. Am J Pathol 177: 2166–2168. 20889567 10.2353/ajpath.2010.100803 PMC2966775

[43] Saito S, Aoki H, Ito A, Ueno S, Wada T, et al (2003) Human alpha2,3-sialyltransferase (ST3Gal II) is a stage-specific embryonic antigen-4 synthase. J Biol Chem 278: 26474–26479. 12716912 10.1074/jbc.M213223200

[44] Gang EJ, Bosnakovski D, Figueiredo CA, Visser JW, Perlingeiro RC (2007) SSEA-4 identifies mesenchymal stem cells from bone marrow. Blood 109: 1743–1751. 17062733 10.1182/blood-2005-11-010504

[45] Miyanishi M, Mori Y, Seita J, Chen JY, Karten S, et al (2013) Do pluripotent stem cells exist in adult mice as very small embryonic stem cells? Stem Cell Reports 1: 198–208. 24052953 10.1016/j.stemcr.2013.07.001 PMC3757755

[46] Ratajczak MZ, Zuba-Surma EK, Machalinski B, Ratajczak J, Kucia M (2008) Very small embryonic-like (VSEL) stem cells: purification from adult organs, characterization, and biological significance. Stem Cell Rev 4: 89–99. 18459073 10.1007/s12015-008-9018-0

[47] Radu A, Pichon C, Camparo P, Antoine M, Allory Y, et al (2010) Expression of follicle-stimulating hormone receptor in tumor blood vessels. N Engl J Med 363: 1621–1630. 20961245 10.1056/NEJMoa1001283

[48] Liu E, Gordonov S, Treiser MD, Moghe PV (2010) Parsing the early cytoskeletal and nuclear organizational cues that demarcate stem cell lineages. Cell Cycle 9: 2108–2117. 20495372 10.4161/cc.9.11.11864

[49] Shibata R, Skurk C, Ouchi N, Galasso G, Kondo K, et al (2008) Adiponectin promotes endothelial progenitor cell number and function. FEBS Lett 582: 1607–1612. 18423403 10.1016/j.febslet.2008.04.006 PMC2435501

[50] Bellows CF, Zhang Y, Chen J, Frazier ML, Kolonin MG (2011) Circulation of progenitor cells in obese and lean colorectal cancer patients. Cancer Epidemiol Biomarkers Prev 20: 2461–2468. 21930958 10.1158/1055-9965.EPI-11-0556 PMC5470315

[51] Chang HH, Hemberg M, Barahona M, Ingber DE, Huang S (2008) Transcriptome-wide noise controls lineage choice in mammalian progenitor cells. Nature 453: 544–547. 18497826 10.1038/nature06965 PMC5546414

[52] Magalhaes DA, Silveira EL, Junta CM, Sandrin-Garcia P, Fachin AL, et al (2006) Promiscuous gene expression in the thymus: the root of central tolerance. Clin Dev Immunol 13: 81–99. 17162352 10.1080/17402520600877091 PMC2270777

[53] Ratajczak MZ, Kucia M, Reca R, Majka M, Janowska-Wieczorek A, et al (2004) Stem cell plasticity revisited: CXCR4-positive cells expressing mRNA for early muscle, liver and neural cells 'hide out' in the bone marrow. Leukemia 18: 29–40. 14586476 10.1038/sj.leu.2403184

[54] Ismail N, Wang Y, Dakhlallah D, Moldovan L, Agarwal K, et al (2013) Macrophage microvesicles induce macrophage differentiation and miR-223 transfer. Blood 121: 984–995. 23144169 10.1182/blood-2011-08-374793 PMC3567345

[55] Quesenberry PJ, Dooner MS, Goldberg LR, Aliotta JM, Pereira M, et al (2012) A new stem cell biology: the continuum and microvesicles. Trans Am Clin Climatol Assoc 123: 152–166. 23303982 PMC3540600

[56] Lewis CE, De PM, Naldini L (2007) Tie2-expressing monocytes and tumor angiogenesis: regulation by hypoxia and angiopoietin-2. Cancer Res 67: 8429–8432. 17875679 10.1158/0008-5472.CAN-07-1684

[57] Rouhl RP, Mertens AE, van Oostenbrugge RJ, Damoiseaux JG, Debrus-Palmans LL, et al (2012) Angiogenic T-cells and putative endothelial progenitor cells in hypertension-related cerebral small vessel disease. Stroke 43: 256–258. 21980212 10.1161/STROKEAHA.111.632208

[58] Harrison DG, Marvar PJ, Titze JM (2012) Vascular inflammatory cells in hypertension. Front Physiol 3: 128 10.3389/fphys.2012.00128.eCollection2012 22586409 PMC3345946

[59] Hur J, Yang HM, Yoon CH, Lee CS, Park KW, et al (2007) Identification of a novel role of T cells in postnatal vasculogenesis: characterization of endothelial progenitor cell colonies. Circulation 116: 1671–1682. 17909106 10.1161/CIRCULATIONAHA.107.694778

[60] Liu X, Li Y, Liu Y, Luo Y, Wang D, et al (2010) Endothelial progenitor cells (EPCs) mobilized and activated by neurotrophic factors may contribute to pathologic neovascularization in diabetic retinopathy. Am J Pathol 176: 504–515. 19948824 10.2353/ajpath.2010.081152 PMC2797908

[61] Cheng S, Wang N, Larson MG, Palmisano JN, Mitchell GF, et al (2012) Circulating angiogenic cell populations, vascular function, and arterial stiffness. Atherosclerosis 220: 145–150. 22093724 10.1016/j.atherosclerosis.2011.10.015 PMC3277804

[62] Huynh J, Nishimura N, Rana K, Peloquin JM, Califano JP, et al (2011) Age-related intimal stiffening enhances endothelial permeability and leukocyte transmigration. Sci Transl Med 3: 112ra122 10.1126/scitranslmed.3002761 PMC3693751 22158860

[63] Horvath S, Zhang Y, Langfelder P, Kahn RS, Boks MP, et al (2012) Aging effects on DNA methylation modules in human brain and blood tissue. Genome Biol 13: R97. 23034122 10.1186/gb-2012-13-10-r97 PMC4053733

[64] Kaess BM, Rong J, Larson MG, Hamburg NM, Vita JA, et al (2012) Aortic stiffness, blood pressure progression, and incident hypertension. JAMA 308: 875–881. 22948697 10.1001/2012.jama.10503 PMC3594687

[65] Oliveras A, de la Sierra A, Martinez-Estrada OM, Larrousse M, Vazquez S, et al (2008) Putative endothelial progenitor cells are associated with flow-mediated dilation in refractory hypertensives. Blood Press 17: 298–305. 19085535 10.1080/08037050802584446

[66] Durik M, Seva PB, Roks AJ (2012) The renin-angiotensin system, bone marrow and progenitor cells. Clin Sci (Lond) 123: 205–223. 22548406 10.1042/CS20110660

[67] De CC, Pilu A, Rizzoni D, Porteri E, Muiesan ML, et al (2011) Effect of antihypertensive treatment on circulating endothelial progenitor cells in patients with mild essential hypertension. Blood Press 20: 77–83. 21114380 10.3109/08037051.2010.535973

[68] Banerji CR, Miranda-Saavedra D, Severini S, Widschwendter M, Enver T, et al (2013) Cellular network entropy as the energy potential in Waddington's differentiation landscape. Sci Rep 3: 3039 10.1038/srep03039 24154593 PMC3807110

