# Correction: A Module of Human Peripheral Blood Mononuclear Cell Transcriptional Network Containing Primitive and Differentiation Markers Is Related to Specific Cardiovascular Health Variables

**DOI:** 10.1371/journal.pone.0104595

**Published:** 2014-07-30

**Authors:** The PLOS ONE Staff

There are two incorrect references to Table 2 in the Results section: the last sentence of the section “Cardiovascular functional validation of genes from Module 1” and the first sentence of the section “Personalized representation of gene analysis.” Both of these instances should refer to Table 3.

There is one incorrect reference to Table 3 in the Results section of the manuscript: the second sentence of “Modifications of PBMC gene expression in hypertensive patients” should refer to Table 2.
