# Supplementary material for: A Module of Human Peripheral Blood Mononuclear Cell Transcriptional Network Containing Primitive and Differentiation Markers Is Related to Specific Cardiovascular Health Variables
Source: PLoS One. 2014 Apr 23;9(4):e95124. doi: 10.1371/journal.pone.0095124 (PMC3997360; doi:10.1371/journal.pone.0095124)
Supplement: Table S1 — Abbreviations: Angio: angiogenesis; EC: endothelial cells; EPC: endothelial progenitor cells; FB: fibroblasts; MC: monocytes; Mph: macrophages; MSC: mesenchymal stem cells; SMC: smooth muscle cells; VSELC: very small embryonic-like stem cells. For some genes, alternative frequently used names are given in parentheses. (DOCX) [file pone.0095124.s001.docx]

| Table S1. Genes analyzed in this study | | |
| --- | --- | --- |
| Gene | **RefSeq ID** | **Description/function [ref.]** |
| Primitive | | |
| ABCG2 | NM_004827 | Side population [1] |
| ALDH1A1 | NM_153329 | Detoxifying/primitive [2] |
| KIT | NM_153329 | Differentiation (CD117) [3] |
| PROM1 | NM_153329 | Differentiation (CD133) [4] |
| CD34 | NM_001773 | Hematopoietic/Progenitor [5] |
| NT5E | NM_002526 | MSC (ecto 5’-nucleotidase; CD73) [6] |
| THY1 | NM_006288 | MSC (CD90) [7] |
| CXCR4 | NM_003467 | Differentiation (SDF-1R) [8] |
| GATA4 | NM_002052 | Early (cardio-) myocyte [9] |
| MKI67 | NM_002417 | Proliferation [3] |
| NANOG | NM_024865 | Differentiation [10] |
| NES | NM_006617 | Neuronal primitive, Angio [11] |
| NOTCH4 | NM_004557 | Differentiation [12] |
| POU5F1 | NM_002701 | Differentiation (Oct4) [10] |
| ST3GAL2 | NM_006927 | SSEA4 synthesis (VSELC) [13,14] |
| Cardiovascular | | |
| ACTA2 | NM_001613 | SMC, FB [15] |
| CAV3 | NM_001234 | (Cardio) myocyte [16] |
| CDH5 | NM_001795 | EC (VE-Cadherin) [17] |
| CNN1 | NM_001299 | SMC, EPC (calponin) [18] |
| COL1A1 | NM_000088 | FB, SMC (collagen 1) [19] |
| CX3CR1 | NM_001337 | SMC/Progenitors [20] |
| FSHR | NM_181446 | Reproduction, Angio [21] |
| KDR | NM_002253 | EC, EPC, HSC (VEGR2) [22] |
| MYH6 | NM_002471 | Cardiomyocyte [23] |
| NKX2-5 | NM_004387 | Early (cardio) myocyte [23] |
| NOS3 | NM_000603 | EC (eNOS) [24] |
| OLR1 | NM_002543 | EC (Lox-1) [25] |
| PECAM1 | NM_000442 | EC (CD31) [26] |
| TEK | NM_000459 | EC, MC (Tie2) [27] |
| VWF | NM_000552 | EC (von Willebrand factor) [28] |
| Other differentiation markers | | |
| ADIPOQ | NM_004797 | Adipocyte (adiponectin) [29] |
| ALB | NM_000477 | Hepatocyte [30] |
| ALPL | NM_000478 | Osteoblast [31] |
| BGLAP | NM_199173 | Osteoblast (osteocalcin) [32] |
| CD14 | NM_000591 | MC, EC [33] |
| CD3E | NM_000733 | Early T cell [34] |
| PTPRC | NM_002838 | Leukocytes (CD45) [35] |
| CD68 | NM_001251 | Mph (MC) [36] |
| CD79A | NM_001783 | Early B cell [37] |
| ENO2 | NM_001975 | Neuronal [38] |
| ITGAM | NM_000632 | MC, Neutrophil [39] |
| KRT14 | NM_000526 | Epithelial (keratin 14) [40] |
| MAP2 | NM_002374 | Neuronal [41] |
| MPO | NM_000250 | Neutrophil/Progenitors [42] |
| SFTBP | NM_000542 | Lung epithelial (lung surfactant) [43] |
| Endogenous controls | | |
| B2M | NM_004048 | Endogenous control |
| GAPDH | NM_002046 | Endogenous control |
| RPL13A | NM_012423 | Endogenous control |

Supplementary References

1 Pfister O, Oikonomopoulos A, Sereti KI, Sohn RL, Cullen D et al. (2008) Role of the ATP-binding cassette transporter Abcg2 in the phenotype and function of cardiac side population cells. Circ Res 103: 825-835.

2 Povsic TJ, Zavodni KL, Kelly FL, Zhu S, Goldschmidt-Clermont PJ et al. (2007) Circulating progenitor cells can be reliably identified on the basis of aldehyde dehydrogenase activity. J Am Coll Cardiol 50: 2243-2248.

3 Orlic D, Kajstura J, Chimenti S, Jakoniuk I, Anderson SM et al. (2001) Bone marrow cells regenerate infarcted myocardium. Nature 410: 701-705.

4 Salven P, Mustjoki S, Alitalo R, Alitalo K, Rafii S (2003) VEGFR-3 and CD133 identify a population of CD34+ lymphatic/vascular endothelial precursor cells. Blood 101: 168-172. 1.

5 Mackie AR, Losordo DW (2011) CD34-positive stem cells: in the treatment of heart and vascular disease in human beings. Tex Heart Inst J 38: 474-485.

6 Kopher RA, Penchev VR, Islam MS, Hill KL, Khosla S et al. (2010) Human embryonic stem cell-derived CD34+ cells function as MSC progenitor cells. Bone 47: 718-728. S8756-3282.

7 De FF, Tirino V, Desiderio V, Ferraro G, D'Andrea F et al. (2009) Human CD34/CD90 ASCs are capable of growing as sphere clusters, producing high levels of VEGF and forming capillaries. PLoS One 4: e6537.

8 Penn MS (2009) Importance of the SDF-1:CXCR4 axis in myocardial repair. Circ Res 104: 1133-1135.

9 Beguin PC, El-Helou V, Assimakopoulos J, Clement R, Gosselin H et al. (2009) The phenotype and potential origin of nestin+ cardiac myocyte-like cells following infarction. J Appl Physiol 107: 1241-1248.

10 Rasini V, Dominici M, Kluba T, Siegel G, Lusenti G et al. (2013) Mesenchymal stromal/stem cells markers in the human bone marrow. Cytotherapy 15: 292-306.

11 Suzuki S, Namiki J, Shibata S, Mastuzaki Y, Okano H (2010) The neural stem/progenitor cell marker nestin is expressed in proliferative endothelial cells, but not in mature vasculature. J Histochem Cytochem 58: 721-730.

12 Dontu G, Jackson KW, McNicholas E, Kawamura MJ, Abdallah WM et al. (2004) Role of Notch signaling in cell-fate determination of human mammary stem/progenitor cells

8. Breast Cancer Res 6: R605-R615.

13 Ratajczak MZ, Zuba-Surma EK, Machalinski B, Ratajczak J, Kucia M (2008) Very small embryonic-like (VSEL) stem cells: purification from adult organs, characterization, and biological significance. Stem Cell Rev 4: 89-99.

14 Saito S, Aoki H, Ito A, Ueno S, Wada T et al. (2003) Human alpha2,3-sialyltransferase (ST3Gal II) is a stage-specific embryonic antigen-4 synthase. J Biol Chem 278: 26474-26479.

15 Ludin A, Itkin T, Gur-Cohen S, Mildner A, Shezen E et al. (2012) Monocytes-macrophages that express alpha-smooth muscle actin preserve primitive hematopoietic cells in the bone marrow. Nat Immunol 13: 1072-1082.

16 Waldenstrom A, Genneback N, Hellman U, Ronquist G (2012) Cardiomyocyte microvesicles contain DNA/RNA and convey biological messages to target cells. PLoS One 7: e34653.

17 Peichev M, Naiyer AJ, Pereira D, Zhu Z, Lane WJ et al. (2000) Expression of VEGFR-2 and AC133 by circulating human CD34(+) cells identifies a population of functional endothelial precursors. Blood 95: 952-958.

18 Long X, Slivano OJ, Cowan SL, Georger MA, Lee TH et al. (2011) Smooth muscle calponin: an unconventional CArG-dependent gene that antagonizes neointimal formation

12. Arterioscler Thromb Vasc Biol 31: 2172-2180.

19 Bucala R, Spiegel LA, Chesney J, Hogan M, Cerami A (1994) Circulating fibrocytes define a new leukocyte subpopulation that mediates tissue repair. Mol Med 1: 71-81.

20 Hung SC, Pochampally RR, Hsu SC, Sanchez C, Chen SC et al. (2007) Short-term exposure of multipotent stromal cells to low oxygen increases their expression of CX3CR1 and CXCR4 and their engraftment in vivo. PLoS One 2: e416.

21 Radu A, Pichon C, Camparo P, Antoine M, Allory Y et al. (2010) Expression of follicle-stimulating hormone receptor in tumor blood vessels. N Engl J Med 363: 1621-1630.

22 Asahara T, Isner JM (2002) Endothelial progenitor cells for vascular regeneration. J Hematother Stem Cell Res 11: 171-178.

23 Ng SL, Narayanan K, Gao S, Wan AC (2011) Lineage restricted progenitors for the repopulation of decellularized heart. Biomaterials 32: 7571-7580.

24 Duda DG, Fukumura D, Jain RK (2004) Role of eNOS in neovascularization: NO for endothelial progenitor cells. Trends Mol Med 10: 143-145.

25 Sawamura T, Kakino A, Fujita Y (2012) LOX-1: a multiligand receptor at the crossroads of response to danger signals. Curr Opin Lipidol 23: 439-445.

26 Liang SX, Khachigian LM, Ahmadi Z, Yang M, Liu S et al. (2011) In vitro and in vivo proliferation, differentiation and migration of cardiac endothelial progenitor cells (SCA1+/CD31+ side-population cells). J Thromb Haemost 9: 1628-1637.

27 Lewis CE, De PM, Naldini L (2007) Tie2-expressing monocytes and tumor angiogenesis: regulation by hypoxia and angiopoietin-2. Cancer Res 67: 8429-8432.

28 Langer HF, der Ruhr JW, Daub K, Schoenberger T, Stellos K et al. (2010) Capture of endothelial progenitor cells by a bispecific protein/monoclonal antibody molecule induces reendothelialization of vascular lesions. J Mol Med 88: 687-699.

29 Xu X, Liu C, Xu Z, Tzan K, Wang A et al. (2012) Altered adipocyte progenitor population and adipose-related gene profile in adipose tissue by long-term high-fat diet in mice. Life Sci 90: 1001-1009.

30 Nava S, Westgren M, Jaksch M, Tibell A, Broome U et al. (2005) Characterization of cells in the developing human liver. Differentiation 73: 249-260.

31 Heinemann DE, Siggelkow H, Ponce LM, Viereck V, Wiese KG et al. (2000) Alkaline phosphatase expression during monocyte differentiation. Overlapping markers as a link between monocytic cells, dendritic cells, osteoclasts and osteoblasts. Immunobiology 202: 68-81.

32 Gossl M, Modder UI, Atkinson EJ, Lerman A, Khosla S (2008) Osteocalcin expression by circulating endothelial progenitor cells in patients with coronary atherosclerosis. J Am Coll Cardiol 52: 1314-1325.

33 Zhang R, Yang H, Li M, Yao Q, Chen C (2005) Acceleration of endothelial-like cell differentiation from CD14+ monocytes in vitro. Exp Hematol 33: 1554-1563.

34 Fischer A, de Saint BG, Le DF (2005) CD3 deficiencies. Curr Opin Allergy Clin Immunol 5: 491-495.

35 Case J, Mead LE, Bessler WK, Prater D, White HA et al. (2007) Human CD34+AC133+VEGFR-2+ cells are not endothelial progenitor cells but distinct, primitive hematopoietic progenitors. Exp Hematol 35: 1109-1118.

36 Krenning G, Dankers PY, Jovanovic D, van Luyn MJ, Harmsen MC (2007) Efficient differentiation of CD14+ monocytic cells into endothelial cells on degradable biomaterials. Biomaterials 28: 1470-1479.

37 Chu PG, Arber DA (2001) CD79: a review. Appl Immunohistochem Mol Morphol 9: 97-106.

38 Oliva D, Cali L, Feo S, Giallongo A (1991) Complete structure of the human gene encoding neuron-specific enolase. Genomics 10: 157-165.

39 Larson RS, Springer TA (1990) Structure and function of leukocyte integrins. Immunol Rev 114: 181-217.

40 Medina A, Brown E, Carr N, Ghahary A (2009) Circulating monocytes have the capacity to be transdifferentiated into keratinocyte-like cells. Wound Repair Regen 17: 268-277.

41 Popescu IR, Nicaise C, Liu S, Bisch G, Knippenberg S et al. (2013) Neural Progenitors Derived From Human Induced Pluripotent Stem Cells Survive and Differentiate Upon Transplantation Into a Rat Model of Amyotrophic Lateral Sclerosis. Stem Cells Transl Med 2: 167-174.

42 Vlasova II, Feng WH, Goff JP, Giorgianni A, Do D et al. (2011) Myeloperoxidase-dependent oxidation of etoposide in human myeloid progenitor CD34+ cells. Mol Pharmacol 79: 479-487.

43 Field-Corbett C, English K, O'Dea S (2009) Regulation of surfactant protein B gene expression in bone marrow-derived cells. Stem Cells 27: 662-669.
