# Supplementary material for: A Module of Human Peripheral Blood Mononuclear Cell Transcriptional Network Containing Primitive and Differentiation Markers Is Related to Specific Cardiovascular Health Variables
Source: PLoS One. 2014 Apr 23;9(4):e95124. doi: 10.1371/journal.pone.0095124 (PMC3997360; doi:10.1371/journal.pone.0095124)
Supplement: Table S2 — (1) In those instances when a gene was represented by several probe sets on the array, we calculated the median signal value for each probe set over all of the arrays and retained the sets that had the highest value. (2) Presence Score was calculated as percentage from all 274 arrays, using MAS5 algorithm (Affymetrix Expression Console). The following GEO datasets were used to generate these data: GSE8507, GSE10041, GSE11761, GSE14642, GSE19743, GSE21942, GSE27034, and GSE46480 (3) Percentages in this column are calculated only from the arrays used in this study [1]. Reference: 1 Li L, Li M, Sun C, Francisco L, Chakraborty S, et al. (2011) Altered hematopoietic cell gene expression precedes development of therapy-related myelodysplasia/acute myeloid leukemia and identifies patients at risk. Cancer Cell 20: 591–605. (GSE23025). (DOCX) [file pone.0095124.s002.docx]

| **Table S2. Detection of select primitive and differentiation genes on Affymetrix GeneChip^®^ microarrays.** | | | | |
| --- | --- | --- | --- | --- |
| **Affymetrix ID^(1)^** | **Gene Symbol** | **RefSeq Transcript ID** | **Presence score^(2)^** | |
|  |  |  | **Normal**  **PBMCs** | **CD34^+(3)^**  **Cells** |
| 208204_s_at | CAV3 | NM_001234 | 0% | 0% |
| 1570276_a_at | GATA4 | NM_002052 | 0% | 0% |
| 209351_at | KRT14 | NM_000526 | 0% | 0% |
| 218678_at | NES | NM_006617 | 0% | 0% |
| 206578_at | NKX2-5 | NM_001166175 | 0% | 0% |
| 217711_at | TEK | NM_000459 | 0% | 0% |
| 213869_x_at | THY1 | NM_006288 | 0% | 0% |
| 211201_at | FSHR | NM_000145 | 0% | 0% |
| 204677_at | CDH5 | NM_001114117 | 0% | 2% |
| 214532_x_at | POU5F1 | NM_001159542 | 0% | 0% |
| 220184_at | NANOG | NM_024865 | 0% | 11% |
| 229093_at | NOS3 | NM_000603 | 0% | 0% |
| 203951_at | CNN1 | NM_001299 | 0% | 9% |
| 212022_s_at | MKI67 | NM_001145966 | 1% | 18% |
| 202112_at | VWF | NM_000552 | 3% | 71% |
| 214837_at | ALB | NM_000477 | 3% | 4% |
| 214468_at | MYH6 | NM_002471 | 5% | 0% |
| 225540_at | MAP2 | NM_001039538 | 7% | 21% |
| 209735_at | ABCG2 | NM_001257386 | 8% | 73% |
| 209543_s_at | CD34 | NM_001025109 | 9% | 88% |
| 203934_at | KDR | NM_002253 | 12% | 16% |
| 207175_at | ADIPOQ | NM_001177800 | 15% | 25% |
| 205051_s_at | KIT | NM_000222 | 20% | 98% |
| 214354_x_at | SFTPB | NM_000542 | 21% | 13% |
| 203949_at | MPO | NM_000250 | 24% | 95% |
| 204304_s_at | PROM1 | NM_001145847 | 28% | 100% |
| 217430_x_at | COL1A1 | NM_000088 | 32% | 39% |
| 210004_at | OLR1 | NM_001172632 | 54% | 89% |
| 205247_at | NOTCH4 | NM_004557 | 54% | 64% |
| 201313_at | ENO2 | NM_001975 | 70% | 91% |
| 215783_s_at | ALPL | NM_000478 | 82% | 36% |
| 203507_at | CD68 | NM_001040059 | 84% | 39% |
| 217650_x_at | ST3GAL2 | NM_006927 | 84% | 96% |
| 206956_at | BGLAP | NM_001199661 | 92% | 84% |
| 212224_at | ALDH1A1 | NM_000689 | 93% | 96% |
| 200974_at | ACTA2 | NM_001141945 | 99% | 98% |
| 205456_at | CD3E | NM_000733 | 99% | 25% |
| 203939_at | NT5E | NM_001204813 | 99% | 55% |
| 205898_at | CX3CR1 | NM_001171171 | 100% | 61% |
| 201743_at | CD14 | NM_000591 | 100% | 88% |
| 205786_s_at | ITGAM | NM_000632 | 100% | 96% |
| 217028_at | CXCR4 | NM_001008540 | 100% | 100% |
| 208982_at | PECAM1 | NM_000442 | 100% | 100% |
| 212587_s_at | PTPRC | NM_001267798 | 100% | 100% |
| 201891_s_at | B2M | NM_004048 | 100% | 100% |
| 212191_x_at | RPL13A | NM_000977 | 100% | 100% |
| 217398_x_at | GAPDH | NM_001256799 | 100% | 100% |
